# Subcellular Distribution of BALF2 and the Role of Rab1 in the Formation of Epstein-Barr Virus Cytoplasmic Assembly Compartment and Virion Release

**DOI:** 10.1128/spectrum.04369-22

**Published:** 2023-01-05

**Authors:** Tsung-Yu Chao, Yi-Ying Cheng, Zi-Yun Wang, Tien-Fang Fang, Yu-Ruei Chang, Chi-Shane Fuh, Mei-Tzu Su, Yuan-Wei Su, Pang-Hung Hsu, Yu-Chen Su, Yu-Ching Chang, Ting-Yau Lee, Wei-Han Chou, Jaap M. Middeldorp, Jaakko Saraste, Mei-Ru Chen

**Affiliations:** a Graduate Institute and Department of Microbiology, College of Medicine, National Taiwan University, Tipei, Taiwan; b Department of Bioscience and Biotechnology, National Taiwan Ocean University, Keelung, Taiwan; c VU University Medical Center, Department of Pathology, Cancer Center Amsterdam, Amsterdam, The Netherlands; d Department of Biomedicine and Molecular Imaging Center, University of Bergen, Bergen, Norway; Wayne State University

**Keywords:** EBV, ER-Golgi transport, virion morphogenesis, nucleocytoplasmic trafficking, single-stranded DNA binding protein, viral tegumentation

## Abstract

Epstein-Barr virus (EBV) replicates its genome in the nucleus and undergoes tegumentation and envelopment in the cytoplasm. We are interested in how the single-stranded DNA binding protein BALF2, which executes its function and distributes predominantly in the nucleus, is packaged into the tegument of virions. At the mid-stage of virus replication in epithelial TW01-EBV cells, a small pool of BALF2 colocalizes with tegument protein BBLF1, BGLF4 protein kinase, and the *cis*-Golgi marker GM130 at the perinuclear viral assembly compartment (AC). A possible nuclear localization signal (NLS) between amino acids 1100 and 1128 (C29), which contains positive charged amino acid ^1113^RRKRR^1117^, is able to promote yellow fluorescent protein (YFP)-LacZ into the nucleus. In addition, BALF2 interacts with the nucleocapsid-associated protein BVRF1, suggesting that BALF2 may be transported into the cytoplasm with nucleocapsids in a nuclear egress complex (NEC)-dependent manner. A group of proteins involved in intracellular transport were identified to interact with BALF2 in a proteomic analysis. Among them, the small GTPase Rab1A functioning in bi-directional trafficking at the ER-Golgi interface is also a tegument component. In reactivated TW01-EBV cells, BALF2 colocalizes with Rab1A in the cytoplasmic AC. Expression of dominant-negative GFP-Rab1A(N124I) diminished the accumulation of BALF2 in the AC, coupling with attenuation of gp350/220 glycosylation. Virion release was significantly downregulated by expressing dominant-negative GFP-Rab1A(N124I). Overall, the subcellular distribution of BALF2 is regulated through its complex interaction with various proteins. Rab1 activity is required for proper gp350/220 glycosylation and the maturation of EBV.

**IMPORTANCE** Upon EBV lytic reactivation, the virus-encoded DNA replication machinery functions in the nucleus, while the newly synthesized DNA is encapsidated and transported to the cytoplasm for final virus assembly. The single-stranded DNA binding protein BALF2 executing functions within the nucleus was also identified in the tegument layer of mature virions. Here, we studied the functional domain of BALF2 that contributes to the nuclear targeting and used a proteomic approach to identify novel BALF2-interacting cellular proteins that may contribute to virion morphogenesis. The GTPase Rab1, a master regulator of anterograde and retrograde endoplasmic reticulum (ER)-Golgi trafficking, colocalizes with BALF2 in the juxtanuclear concave region at the midstage of EBV reactivation. Rab1 activity is required for BALF2 targeting to the cytoplasmic assembly compartment (AC) and for gp350/220 targeting to *cis*-Golgi for proper glycosylation and virion release. Our study hints that EBV hijacks the bi-directional ER-Golgi trafficking machinery to complete virus assembly.

## INTRODUCTION

Epstein-Barr virus (EBV) is a ubiquitous gamma herpesvirus that infects the majority of people worldwide. The virion is composed of a 172-kb double-stranded DNA within the icosahedral capsid, the host cell-derived lipid membrane bilayer containing multiple viral glycoproteins, and a protein-containing tegument layer in between. The viral and cellular proteins in the tegument are believed to be required for the next round infection (1). EBV mainly infects B lymphocytes and epithelial cells via binding to specific receptors. After receptor binding and viral glycoprotein-mediated membrane fusion, the nucleocapsid is translocated close to the nuclear pores, and the viral genome is injected into the nucleus. The linear viral DNA is circularized through the terminal repeats (TR) into an episomal form and maintained latently in the cell by EBNA-1 tethering to host metaphase chromosome during cell division (2). Latent EBV can be reactivated into lytic replication through various signaling pathways, including histone deacetylase inhibitors, PKCδ activators, or direct expression of immediate early transactivators Zta or Rta (3–6).

Lytic EBV DNA replication is carried out by a viral DNA replication complex which contains eight virus-encoded proteins, BZLF1 (oriLyt-binding protein), BRLF1 (immediate early transactivator), BALF5 (DNA polymerase), BMRF1 (DNA polymerase processivity factor), BALF2 (single-stranded DNA-binding protein), BBLF4 (helicase), BSLF1 (primase), and BBLF2/3 (helicase-primase-associated protein) (7, 8). In addition, we found that EBV BKRF3-encoded viral uracil DNA glycosylase (UDG) contributes to EBV DNA replication through physical interactions with proteins in the DNA replication complex (9, 10). Intriguingly, by using sophisticated isotope-coded affinity tag (ICAT) analysis to distinguish tegument proteins from envelope and capsid proteins, the DNA replication complex-associated BMRF1 and BALF2, and BGLF4 protein kinases were also identified as virion tegument proteins by mass spectrometry analysis (1).

The nuclear targeting of EBV DNA replication proteins involves modification of nucleocytoplasmic transport and regulation of noncanonical nuclear targeting. For example, we demonstrated that EBV uracil DNA glycosylase BKRF3 does not contain a canonical nuclear localization signal (NLS) but is translocated into the nucleus through its interaction with viral DNA polymerase processivity factor BMRF1 (10). In addition, we found that BGLF4 kinase is imported into the nucleus through its direct interaction with phenylalanine-glycine (FG) nucleoporins in an importin β-independent pathway (11). Simultaneously, BGLF4 also promotes via unknown mechanisms the nuclear import of other DNA replication proteins lacking canonical NLS, including primase, helicase, and primase-associated factors and viral capsid protein BcLF1 (11).

Similar to herpes simplex virus 1 (HSV-1) and human cytomegalovirus (HCMV), after viral DNA replication and encapsidation within the nucleus, the EBV nucleocapsids are exported from the nucleus through the function of nuclear egress complex (NEC) BFRF1/BFLF2. Viral tegumentation then occurs in the juxtanuclear region where the Golgi apparatus resides. Multiple cellular and viral proteins required for the initiation of the next round of infection are recruited into the tegument layer, and their specific interactions with proteins of the Golgi-derived viral envelopes are required for final envelopment and the formation of mature virions. Tegument proteins may be involved in targeting of viral DNA to the nucleus in the next round of infection, recruitment of cellular molecular motors, regulation of viral and host cellular gene and protein expression, and/or assembly of virions during egress (12, 13). It has also been suggested that specific tegument proteins (e.g., BNRF1 of EBV or ICP0 of HSV-1) may couple with viral immediate early genes (BZLF1 of EBV or newly synthesized ICP0 of HSV-1) to eliminate the host defense system component protein ND10 for enhancing the infection efficiency (14).

Studies of alpha-herpesvirus HSV-1 suggested that the tegumentation process may be separated into two distinct steps. The inner tegument proteins may attach to the nucleocapsids in the nucleus before their nuclear egress. The outer tegument proteins may be incorporated during the proposed budding into the *trans*-Golgi network (TGN) or possibly associate with the post-Golgi vesicles bound to the cytoplasmic tails of viral envelope proteins (15). By using different concentrations of KCl to dissociate different layers of the HSV virion structure, UL37p (homolog of EBV BOLF1), UL36p (EBV BPLF1), and US3 protein kinase were found as the most abundant inner tegument proteins (16). Each viral capsid contains 12 pentagram-shaped capsid vertex-specific components (CVSCs), which are composed of a UL17 monomer, a UL25 (homolog of EBV BVRF1) dimer, and a UL36 dimer of the capsid. The CVSCs function as hubs for protein-protein interactions that are important for capsid assembly and for the package of viral genome in the nucleus. In addition, UL36 also provides a foundation for the recruitment of outer tegument in the cytoplasm; it binds the outer tegument protein UL48p (VP16), which in turn connects to UL46p (VP11/12), UL47p (VP13/14), UL49p (VP22), and membrane-embedded envelope proteins (15, 17). Nevertheless, capsid final envelopment is coupled with completion of the outer tegument and incorporation of envelope proteins into the mature virion simultaneously at the cytoplasmic assembly compartment.

Knowledge regarding the specific regulation of the sorting of EBV proteins during tegumentation is still limited. A recent study of B cells revealed that EBV acquires its final envelope at intracellular membranes containing Golgi markers (18). Protein-protein interaction analysis found that the capsid-associated BGLF2 interacts with BBLF1, which is a myristoylated viral tegument protein, possibly facilitating association of the tegumented capsids with viral glycoprotein-embedded Golgi membranes during viral budding (19). Our study of the structure and components of the EBV cytoplasmic assembly compartment also showed that cellular organelle membranes are highly reorganized into a distinct compartment in the juxtanuclear region at the midstage of lytic replication, suggesting that the nuclear egress process directly links to the cytoplasmic assembly compartment (Y.C. Dai et al., manuscript in preparation).

In this study, our aim is to investigate how the subcellular localization of EBV single-stranded DNA binding protein BALF2 is regulated and how viral proteins with functions in the nucleus are incorporated into the virion. BALF2 with a predicted size of 123 kDa is an essential replication factor stabilizing the single-stranded DNA at the viral DNA replication fork during genome replication and forming complexes with DNA polymerase and helicase in the nucleus. Intriguingly, BALF2 is packaged in the tegument layer of mature virions (1). On the other hand, BALF2 was found by yeast two-hybrid screening to interact with GM130, a *cis*-Golgi protein that acts as a tethering factor in endoplasmic reticulum (ER)-Golgi vesicle trafficking, and other cellular cytoplasmic proteins, including zyxin, which can regulate actin fiber reorganization and gene expression by shuttling from the focal adhesion complex to the nucleus (20, 21). It thus suggests that BALF2 may have additional functions other than maintaining the viral DNA replication fork during EBV replication. Here, we examine the nuclear targeting mechanism of BALF2 and further explore its function using a proteomic approach to identify BALF2-interacting viral and cellular factors that contribute to the viral tegumentation and maturation processes.

## RESULTS

### A portion of BALF2 localizes to the juxtanuclear concave Golgi region of epithelial TW01-EBV cells after Rta induction of the lytic cycle.

**FIG 1:**
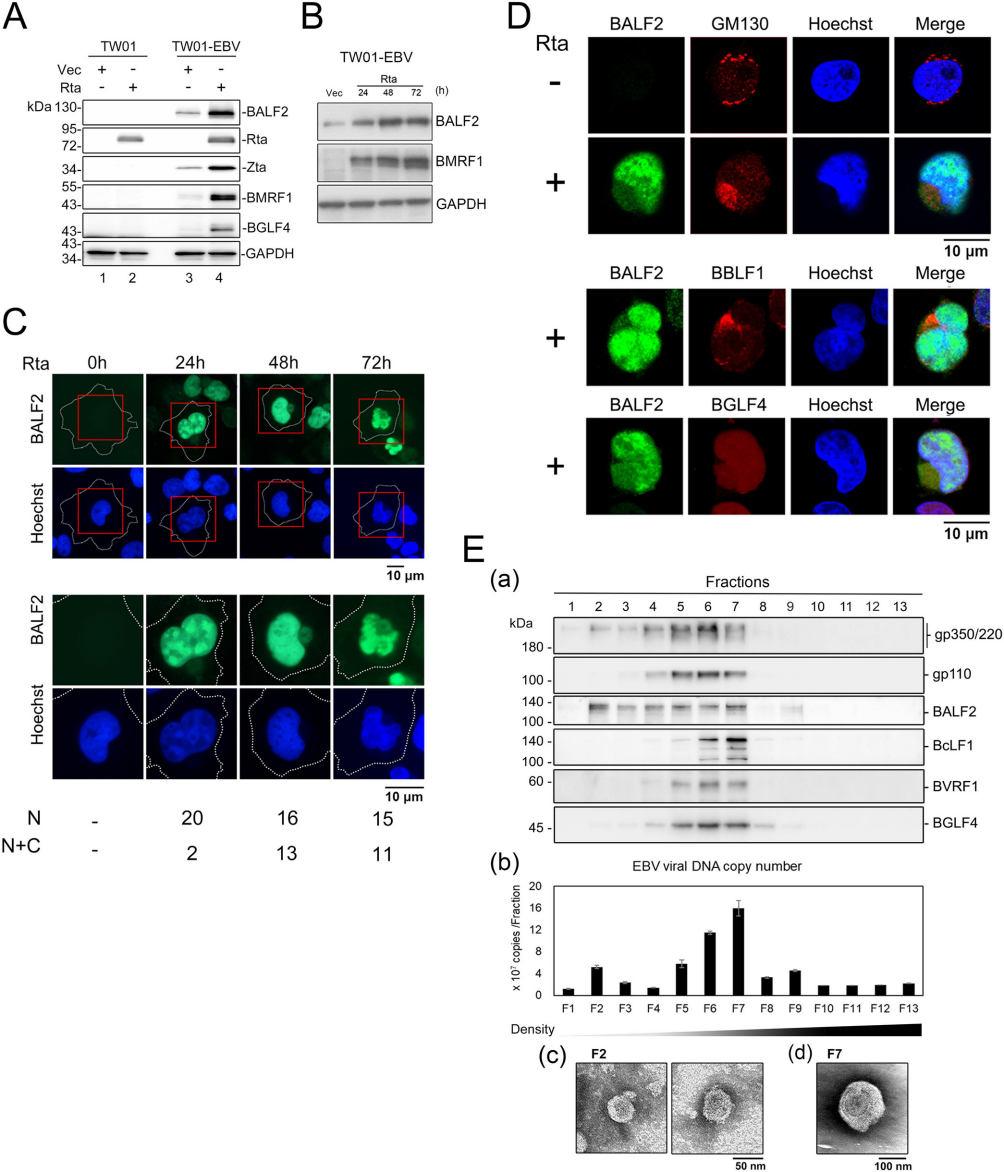
The majority of BALF2 is detected in the nucleus, and a small portion of BALF2 localizes to the juxtanuclear concave region and colocalizes with assembly compartment components at the mid-stage of EBV reactivation in TW01-EBV cells. (A) The specificity of BALF2 monoclonal antibody (OT13B) was examined by immunoblotting of pSG5-Rta or pSG5 vector-transfected TW01-EBV cell lysates. A similar setting of transfected cell lysates of the parental TW01 cells served the negative control. BRLF1 (Rta) and BMRF1 (EA-D) expression was detected as lytic markers. GAPDH served as the loading control. The detection of slight signals of BMRF1 and BALF2 indicates that a small portion of TW01-EBV underwent spontaneous lytic reactivation. (B) The BALF2 expression levels were examined at 24, 48, and 72 h post-pSG5-Rta transfection into TW01-EBV cells. GAPDH served as the loading control. (C) The distribution of BALF2 in TW01-EBV cells at 24, 48, and 72 h post-Rta transfection was detected by OT13B Ab and observed with confocal microscopy. Cell numbers with nuclear only (N) or partial cytoplasmic (N+C) distribution of BALF2 are indicated at the bottom. (D) The subcellular distribution of BALF2 and components in the cytoplasmic assembly compartment in TW01-EBV cells was detected by immunofluorescence staining and observed by confocal microscopy. The slide-cultured TW01-EBV cells were harvested at 48 h post-pSG5-Rta or pSG5 transfection and stained with specific antibodies. BALF2 (green) was detected together with *cis*-Golgi marker GM130 (top panel), EBV tegument protein BBLF1 (middle panel), or BGLF4 (bottom panel). (E) EBV virions were purified from TPA/sodium-induced B95-8 cells and subjected to a sucrose gradient fractionation. (a) Virion components, including BLLF1 (gp350/220), BcLF1 (VCA), BALF2 (single-stranded DNA [ssDNA] binding protein), BVRF1 (capsid associated tegument protein, DNA packaging factor), and BGLF4 (protein kinase), were detected by immunoblotting with specific antibodies. (b) EBV genomic DNA was detected by qPCR amplification of BALF5 in each fraction (bottom panel). (c and d) Fractions F2 and F7 from panel a were fixed and stained with 1% uranyl acetate and then observed under TEM at 200 kV and ×12,000 magnification. In F2, the size of vesicle-like particles is small (~50 nm diameter). In F7, the virion size is around ~160 nm.

To explore how the nucleus-distributed EBV proteins are packaged into the tegument of mature virions, we monitored the subcellular distribution of BALF2 during viral lytic replication. We considered that the advantage of a large cytoplasmic space of epithelial cells may provide better resolution of protein distribution during EBV replication. Therefore, the immediate early transactivator Rta expression plasmid was transfected into EBV-positive epithelial TW01-EBV cells which were derived from recombinant Akata EBV-converted NTUTW01 cells. The specificity of monoclonal anti-BALF2 antibody OT13B was first confirmed in TW01-EBV cells by Western blotting, showing detection of the expected band of 130 kDa at 48 h post-Rta-mediated EBV reactivation (Fig. 1A). Weak signals of BALF2 and BMRF1 were detected in vector-transfected TW01-EBV cells but not in NTUTW01 (TW01) cells, suggesting that a small portion of TW01-EBV cells may undergo spontaneous lytic replication (Fig. 1A). In a kinetic analysis, BALF2 was detectable at 24 h posttransfection (hpt) and increased at 48 and 72 hpt (Fig. 1B). By confocal microscopic analysis, we found that ca. 90% of cells show nuclear expression of BALF2 at 24 hpt, while additional BALF2 signals in the perinuclear concave region were detected in 45% of cells at 48 hpt and 42% of cells at 72 hpt (Fig. 1C). At 48 hpt, the cytoplasmic staining of BALF2 predominantly localized to the concave region limited by the kidney-shaped nucleus, which is very similar to the cytoplasmic assembly compartment (AC) described in cytomegalovirus-infected cells (22). At 72 hpt, the nuclear morphology becomes irregular, coupling with the less compact staining of cytoplasmic BALF2 at AC and coinciding with the release of viruses from the cells (data not shown). We suggest that a small portion of BALF2 may be transported into the cytoplasm through association with nucleocapsids to the cytoplasmic AC. Because it was reported that EBV matures at Golgi-related membranes (18, 23), we costained cells for BALF2 and *cis*-Golgi network marker GM130 and the EBV tegument protein BBLF1 to examine if BALF2 translocated to the Golgi membrane-associated compartment (Fig. 1D). Indeed, a portion of BALF2 colocalized with GM130, the cytoplasmic tegument protein BBLF1, and BGLF4 protein kinase at the concave juxtanuclear Golgi GM130 staining region (Fig. 1D, upper and middle panels), which is very similar to the viral AC described in HCMV-infected cells (22). Our unpublished data also found that the AC contains Golgi, and ER markers, suggesting that this compact region is the viral cytoplasmic assembly compartment (Y.C. Dai et al., manuscript in preparation). The results suggest that BALF2 is translocated into the cytoplasm from the nucleus at the mid- to late stage of EBV replication, possibly for its tegumentation into the virion. Furthermore, we also observed the colocalization of BALF2 with viral protein kinase BGLF4, which is also an intranuclear and a tegument protein (1, 24). Data here suggest that BALF2 with a nuclear distribution is possibly recruited to the AC through protein-protein interaction.

To confirm the incorporation of BALF2 and BGLF4 within the virion, we purified EBV particles from tetradecanoyl phorbol acetate (TPA)/sodium butyrate-induced B95-8 cells, which is a marmoset cell line that produce EBV efficiently, according to our previous protocol (24). After a sucrose gradient sedimentation, viral proteins, including glycoprotein gp350/220 (BLLF1), gp110 (BALF4), viral capsid VCA (BcLF1), BVRF1, BGLF4 kinase, and BALF2, were detected in Western blotting, and the viral DNA was detected by quantitative PCR (qPCR) to indicate the distribution of virions in each fraction (Fig. 1E, panels a and b). BGLF4 kinase, major capsid protein BcLF1, gp110, and the capsid-associated tegument protein BVRF1 were detected at fractions 4 to 7 and displayed a peak distribution in fraction 7 (Fig. 1E, panel a). In addition to signals detected in fractions 4 to 7, BALF2 was cofractionated with gp350/220 and a small amount of viral DNA in fraction 2. Thus, fractions 2 and 7 were then observed under transmission electron microscopy (TEM) with uranyl acetate staining. The micrographs showed vesicle-like structures with diameters around 50 nm in F2 (Fig. 1E, panel c), whereas virus particles (around 160 nm) with the capsid in the center and were observed in F7 (Fig. 1E, panel d). Data thus suggested that BALF2 colocalizes with other viral tegument proteins at the cytoplasmic assembly compartment in TW01-EBV cells and is incorporated into mature virions. The cofractionation of BALF2 and gp350/220 may be incorporated into those smaller defective vesicles through an unknown mechanism.

### The amino acid motif ^1113^RRKRR^1117^ promotes nuclear targeting of BALF2.

**FIG 2:**
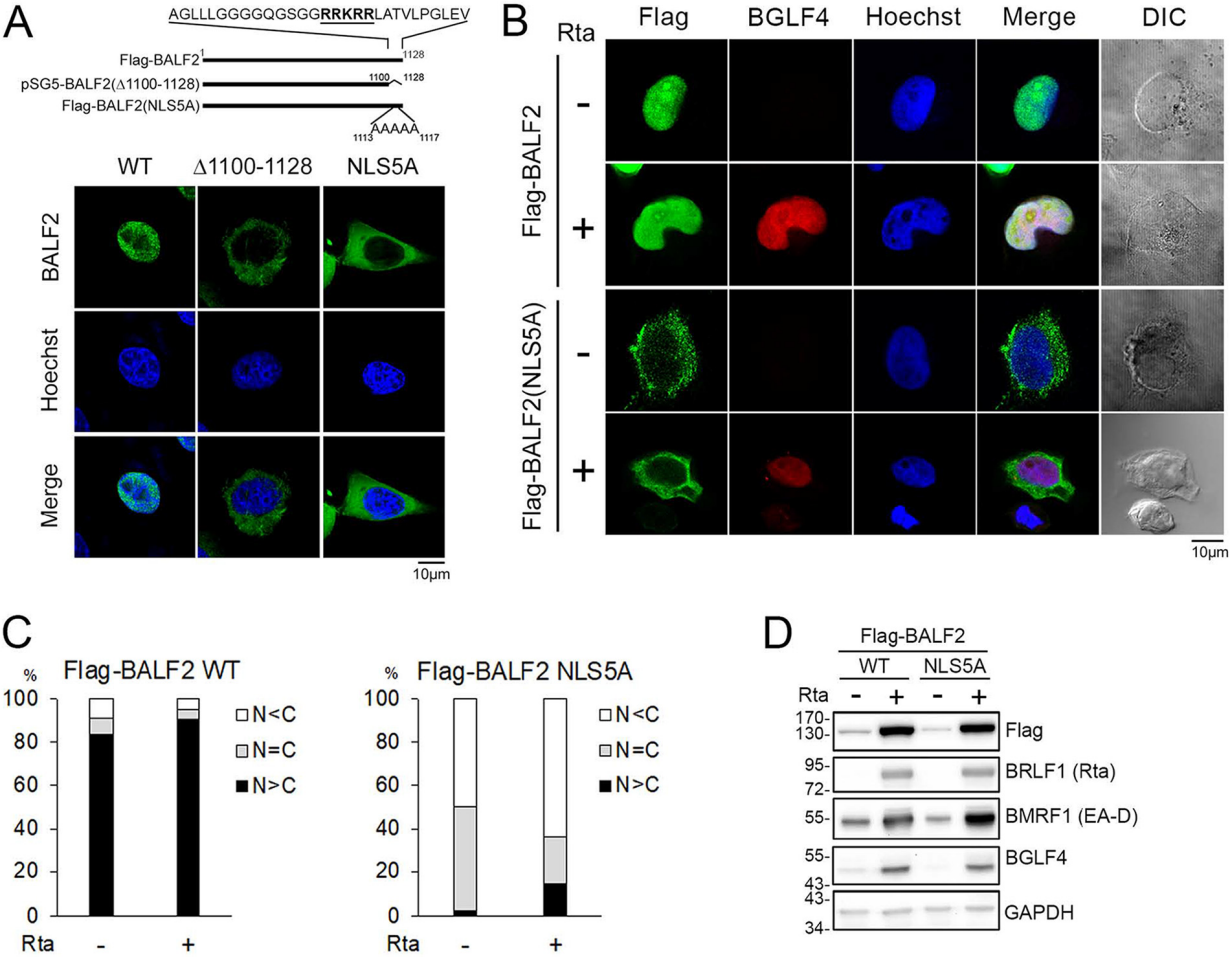
The C terminus basic amino acids of BALF2 are crucial for nucleus localization. (A) WT BALF2 encodes 1,128 amino acids, while BALF2Δ1100-1128 indicates the deletion of 29 C-terminal amino acids, and BALF2(NLS5A) indicates ^1113^RRKRR^1117^ mutation to 5 alanines. HeLa cells were transfected with pCMV-Flag-BALF2, pSG5-BALF2 ΔNLS (Δ1100-1128), or Flag-BALF2(NLS5A). The slides were harvested at 24 h posttransfection, and the distribution of BALF2 was detected with OT13B and observed under confocal microscopy. (B) TW01-EBV cells were cotransfected with pSG5 or pSG5-Rta plus Flag-BALF2 or Flag-BALF2(NLS5A). The slides were harvested at 48 h posttransfection, and the distribution of wild-type Flag-BALF2 or Flag-BALF2(NLS5A) was detected with anti-Flag antibody and observed with confocal microscopy. Viral BGLF4 kinase was also detected with monoclonal Ab 2224 to indicate the lytic cycle progression and the kidney shape of the nucleus. (C) The percentages of cells showing various subcellular distributions of BALF2 in panel B are shown as bar charts. (D) Expression levels of Flag-BALF2 WT and Flag-BALF2(NLS5A) in panel B were analyzed by immunoblotting. After transfection of Rta-expressing plasmid, both Flag-BALF2 WT and Flag-BALF2(NLS5A) expression levels increased, possibly due to stabilization by other viral proteins, while the BMRF1 and BGLF4 expression levels were not affected by Flag-BALF2 or Flag-BALF2(NLS5A). GAPDH served as the loading control.

Because several proteins within the EBV DNA replication compartment do not contain canonical nuclear localization signals, we then examined the nuclear targeting control of BALF2. Within the C terminus, a stretch of 5 positively charged amino acids was identified between amino acids 1113 and 1117. We generated a carboxyl terminus deletion, BALF2Δ(1100-1128), to clarify the contribution of ^1113^RRKRR^1117^ in nuclear translocation of BALF2. In addition, all 5 positively charged amino acids were mutated into alanines for the mutant designated BALF2(NLS5A). Similar to BALF2(Δ1100-1128), BALF2(NLS5A) was distributed predominantly to the cytoplasm (Fig. 2A), suggesting that ^1113^RRKRR^1117^ is the nuclear localization signal (NLS) of BALF2. Because some EBV DNA replication-associated proteins are transported into the nucleus by interacting with other NLS-containing viral proteins, we also cotransfected pCMV-Flag-BALF2 or pCMV-Flag-BALF2(NLS5A) with a control vector or the pSG5-Rta plasmid into EBV-positive TW01-EBV cells and monitored whether Flag-BALF2(NLS5A) can be translocated into the nucleus upon lytic cycle progression as indicated by the detection of viral protein kinase BGLF4. The results showed that Flag-BALF2 is continuously expressed in the nucleus of TW01-EBV cells, whereas Flag-BALF2(NLS5A) is retained in the cytoplasm, even during Rta-induced lytic cycle progression (Fig. 2B and C). Thus, the nuclear targeting of BALF2 requires ^1113^RRKRR^1117^, even in the presence of other viral proteins (Fig. 2D).

### Nuclear targeting of BALF2 is importin-β dependent.

**FIG 3:**
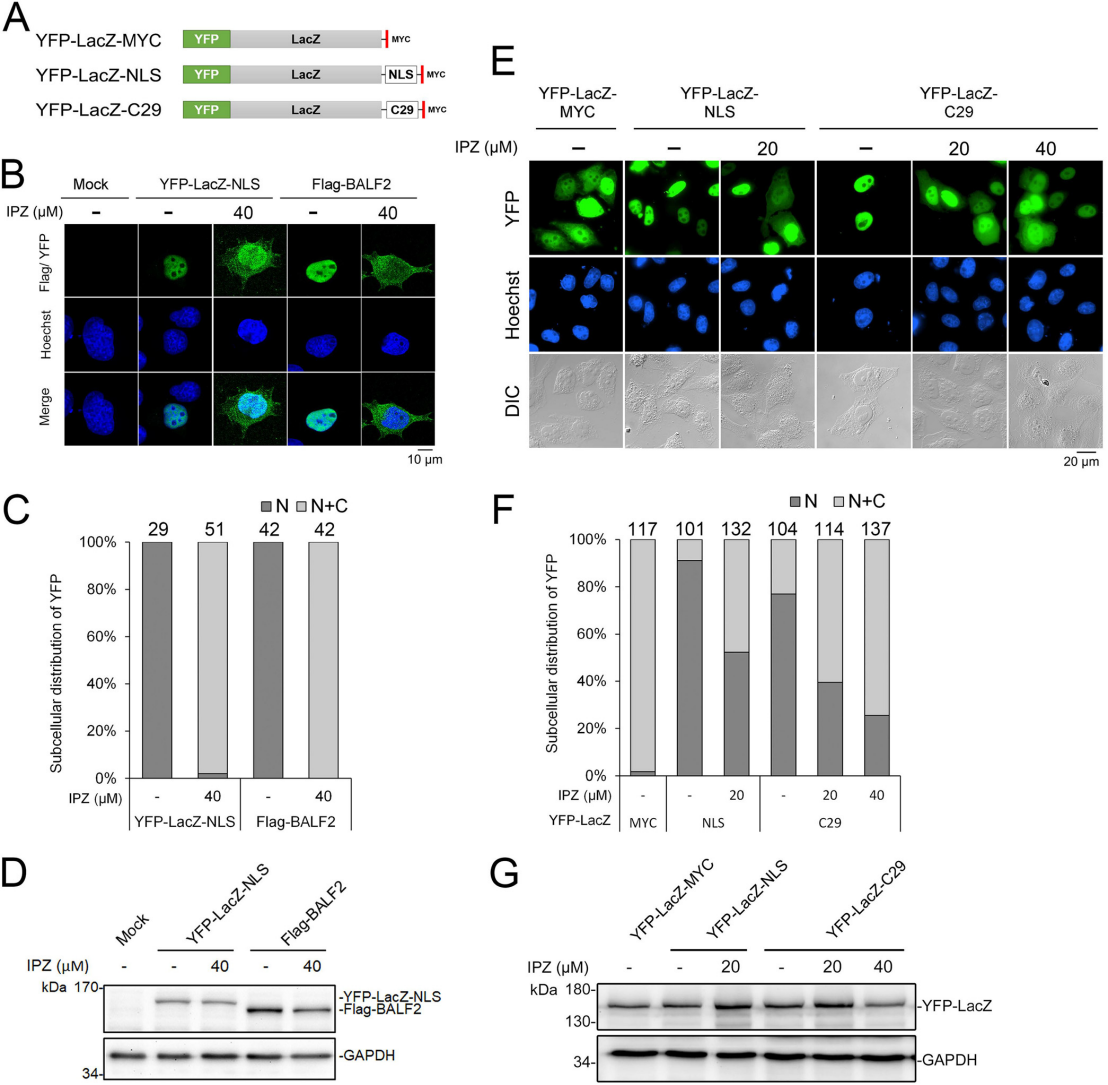
BALF2 nuclear localization is importin-β dependent, and the carboxyl terminal 29 amino acids (aa) promote nuclear targeting of YFP-LacZ. (A) Schematic diagram of YFP-LacZ-fusion proteins with Myc-tag, NLS of SV40 T antigen, or 29 C-terminal amino acids of EBV BALF2. (B) Slide-cultured HeLa cells were transfected with YFP-LacZ-NLS or Flag-BALF2. At 6 h posttransfection, cells were treated with dimethyl sulfoxide (DMSO) or IPZ (40 μM). The slides were harvested at 24 h posttransfection. The distribution of BALF2 was detected with anti-Flag antibody and observed by confocal microscopy. (C) The quantified result of panel B is displayed as a 100% stacked column chart. (D) The expression levels of YFP-LacZ-NLS and Flag-BALF2 in panel B were analyzed by immunoblotting. GAPDH served as the loading control. (E) In the fluorescence microscopy analysis, YFP-LacZ-MYC diffused in the whole cell (115/117), whereas YFP-LacZ-NLS localized in the nucleus of most cells (92/101). YFP-LacZ-C29 predominantly distributed in the nucleus (80/104). In the presence of 20 μM importin-β inhibitor IPZ, 60.5% of YFP-LacZ-C29 expressing-cells displayed partial cytoplasm retention of YFP signals. The YFP-LacZ-fusion protein distribution patterns with or without IPZ treatment were analyzed. (F) The quantified data are showed in a stacked column chart. (G) Expression levels of YFP-LacZ-fusion proteins were analyzed by immunoblotting.

To further examine whether BALF2 nuclear targeting occurs through the importin-β-dependent mechanism, we took advantage of the importin-β inhibitor importazole (IPZ), which blocks the interaction between importin-β and Ran-GTP. A yellow fluorescent protein (YFP)-LacZ-NLS control (25), which contains the SV40 large T antigen nuclear localization signal, was also included for IPZ function control (Fig. 3A). The protein expression levels were similar in the presence or absence of 40 μM IPZ. The nuclear targeting of both YFP-LacZ-NLS and Flag-BALF2 were attenuated by IPZ treatment, suggesting that the nuclear targeting of BALF2 occurs through an importin-β-dependent pathway (Fig. 3B to D).

### The C-terminal ^1113^RRKRR^1117^ of BALF2 can be transferred onto YFP-LacZ to promote nucleus targeting.

To further characterize whether amino acids 1110 to 1128 are sufficient for nuclear targeting, the C-terminal 29 amino acids AGLLLGGGGQGSGG**RRKRR**LATVLPGLEV (C29 in short) were fused to an NLS reporter YFP-LacZ-expressing plasmid. The construct was designated YFP-LacZ-C29 (Fig. 3A). In transiently transfected HeLa cells, the YFP-LacZ-C29 pattern was analyzed and compared to that of YFP-LacZ-MYC and YFP-LacZ-NLS, (Fig. 3A, E, and F). Most cells expressing YFP-LacZ-NLS showed nuclear distribution of YFP signals (92/101), whereas 98.2% (115/117) of the YFP-LacZ-MYC-positive cells displayed a diffuse pattern in both nucleus and cytoplasm. The fusion of C29 of BALF2 promoted the shift of the YFP signals into the nucleus (80/104), suggesting that C29 alone is sufficient for nuclear targeting (Fig. 3E and F). In the presence of 20 μM or 40 μM importin-β inhibitor IPZ, 60.5% (69/114) and 74.5% (102/137) of YFP-LacZ-C29-expressing cells displayed cytoplasmic retention of BALF2-C29-tagged YFP-LacZ. This result indicated that the C-terminal 29 amino acids is critical for BALF2 nuclear localization in an importin-β-mediated manner. Similar protein expression levels were revealed by immunoblotting (Fig. 3G).

### BALF2 interacts with nucleocapsid-associated protein BVRF1.

**FIG 4:**
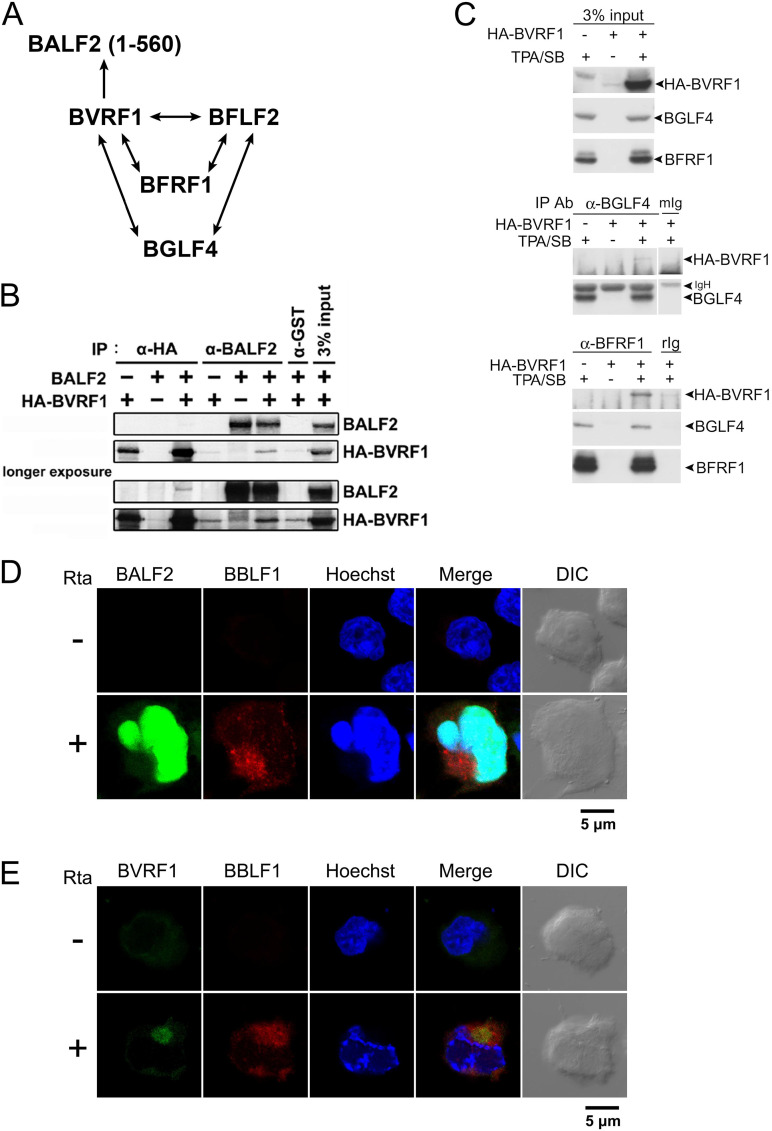
Interactions of BALF2 and BVRF1 are identified in yeast two-hybrid screening and confirmed in coimmunoprecipitation. (A) The diagram depicts the interactions between BGLF4, BFRF1, BFLF2, BVRF1, and BALF2 (amino acids 1 to 560) that were identified in a yeast two-hybrid screen. (B) Coimmunoprecipitation of BALF2 and HA-BVRF1 using antibodies against hemagglutinin (HA), BALF2, and glutathione *S*-transferase (GST; negative control) was carried out with HEK293T cell lysates harvested at 48 h post-cotransfection of BALF2 and HA-BVRF1-expressing plasmids. Immunoblotting was detected with BALF2 antibody and HA antibody. (C) TW01-EBV cells were transfected with HA-BVRF1 for 4 h and treated with TPA (40 ng/mL)/sodium butyrate (3 mM) for 72 h. The immunocomplexes pulled down by BGLF4 antibody (2224) or BFRF1 monoclonal Ab (a gift from Alberto Faggioni, Università La Sapienza, Italy) were analyzed by Western blotting with HA antibody (HA.11), BGLF4 antibody (2616), or BFRF1 Ab. (D and E) TW01-EBV cells were seeded on nonfluorescence slides (7 × 10^5^ cells/10-cm dish) and then cultured overnight and transfected with pSG5 or pSG5-Rta for induction. At 48 hpt, confocal images of the distribution of BALF2 (D) and BVRF1 (E) were detected together with tegument protein BBLF1. The primary antibodies used were BALF2 (OT13B, mouse) at a 1:800 dilution, BVRF1 (mouse) at 1:100, and BBLF1 (rabbit) at 1:100. Secondary antibodies were FITC conjugated anti-mouse IgG (1:100) and Rhodamine conjugated anti-rabbit IgG (1:100).

We were then interested in identifying the BALF2-interacting viral proteins that may facilitate its incorporation into virions. In a previous single gene-to single gene-based yeast two-hybrid screening of viral protein-protein interaction conducted by our collaborator Hsiu-Ming Shih (at Academia Sinica, Taiwan), BALF2 was found to interact with the EBV capsid-associated tegument protein BVRF1, which was proposed to help stabilize the genome packaging into the procapsid. The BVRF1-interacting proteins identified in the yeast two-hybrid screening are summarized in Fig. 4A. Here, the interaction was confirmed by the reciprocal detection of BALF2 and HA-BVRF1 in the immunocomplexes from the 293T-transiently transfected lysates (Fig. 4B). In addition, we also confirmed BVRF1-interacting proteins identified in yeast two-hybrid screening by coimmunoprecipitation of HA-BVRF1 transiently transfected TW01-EBV lysates, including EBV nuclear egress complex (NEC) component BFRF1 and tegument protein BGLF4 kinase (Fig. 4C). In the confocal analysis, we were not able to detect BALF2 and BVRF1 at the same time because both antibodies were derived from mouse. Nevertheless, we observed that a portion of BALF2 was distributed to the center of the tegument protein BBLF1 cluster region (Fig. 4D); similarly, BVRF1 was also detected in the same region at 48 h post-Rta transfection (Fig. 4E). We propose that BALF2 and BGLF4 may be transported from the nucleus to the cytoplasm through the interaction with the BVRF1-mediated complex and NEC complexes.

### BALF2 interacts with the cellular vesicular trafficking machinery.

As described in Fig. 1, at the mid- to late stage of EBV lytic replication in TW01-EBV cells, a minor fraction of BALF2 was translocated from the nucleus to the cytoplasm, occupying the juxtanuclear concave region, where the cytoplasmic AC is expected to reside. To further explore the cellular factors involved in the cytoplasmic distribution of BALF2, TW01-EBV cells were transfected with Flag-BALF2 and Rta-expressing plasmids for reactivation. The cell lysates were harvested at 48 h post-Rta transfection and subjected to immunoprecipitation against Flag followed, by mass spectrum analysis. The proteins detected in Flag-vector-transfected cells were subtracted from those identified in Flag-BALF2-transfected cells. The remaining targets were subjected to analysis using the STRING algorithm (https://string-db.org/).

**FIG 5:**
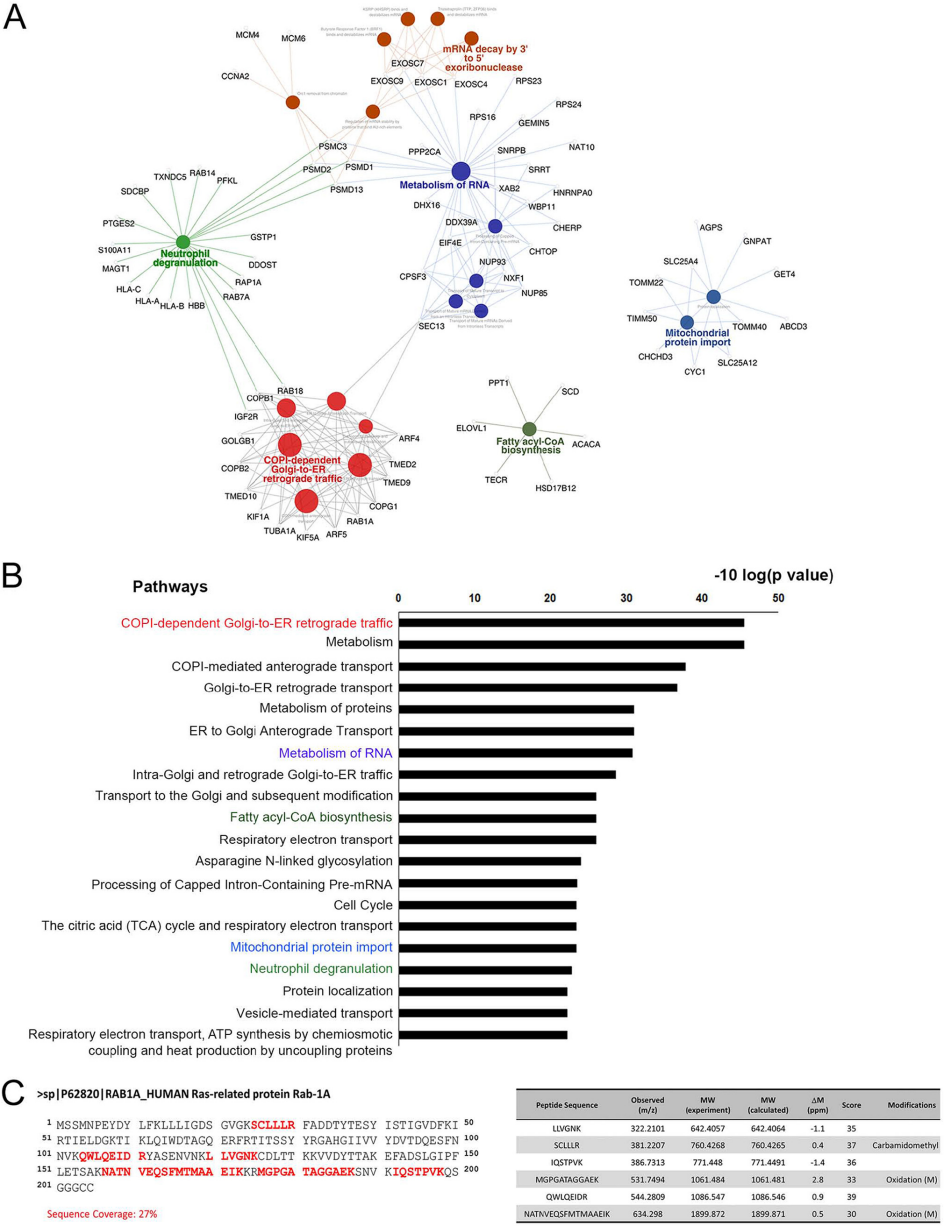
The BALF2 interactome is identified by mass analysis. (A) TW01-EBV cells were transfected with Rta and Flag-BALF2- or Flag-Tag-2B-expressing plasmid. Immunoprecipitation against Flag was carried out at 48 h posttransfection. The BALF2-interacting proteins (*N* = 291 proteins) were defined as proteins identified in the Flag-Tag-2B group subtracted from those in the Flag-BALF2 group. The BALF2 protein-protein interaction (PPI) network was constructed using STRING database v11.0 and displayed using Cytoscape v3.8.0 with the ClueGO app. The represented pathways were enriched using the Reactome database with a Benjamini-Hochberg adjusted *P* value of <0.05. (B) Pathway enrichment analysis of the BALF2 interactome. The *x* axis represents the *P* value, and the *y* axis is the top 20 pathways significantly enriched in BALF2 PPIs. (C) The sequence coverage of Rab1A and scores are indicated.

The proteins involved in pathways identified in the Reactome Pathway Database with a *P* value of <0.05 were labeled on the Flag-BALF2-interacting protein-associated network (Fig. 5A). In addition to the expected proteins involved in gene expression, DNA replication, and the cell cycle, several viral proteins, including BBLF2/BBLF3 (viral primase-associated factor), BORF2/BaRF1 (ribonucleotide reductase), BALF5 (viral DNA polymerase), BSLF1 (viral primase), BMLF1, BRLF1, BVRF2, BXRF1, BMRF1, EBNA1, and LF3 were also pulled down by BALF2. Interestingly, a large group of the proteins identified fell under the category “coat protein complex I (COPI)-dependent Golgi-to-ER retrograde traffic or COPI-mediated anterograde transport,” whereas some proteins are known to be involved in neutrophil degranulation, RNA metabolism, or mitochondrial protein import. The top 20 Flag-BALF2-involved pathways are listed in Fig. 5B in the order of lower false-discovery rate. Notably, the pathway analysis revealed that BALF2 interacts with proteins participating in the COPI-dependent trafficking at the ER-Golgi boundary, which is regulated by Rab1 (26, 27). Rab1 belongs to the Rab small GTPase family with two isoforms, Rab1A and Rab1B. Similar to other Rab proteins, Rab1 cycles between a GDP-bound state (inactive form) and a GTP-bound state (active form), based on the activities of GTPase-activating proteins (GAPs) and GDP/GTP exchange factors (GEFs). With 93% similarity on amino acid sequence, functions between Rab1A and Rab1B in bidirectional ER-Golgi intermediate vesicle trafficking is indistinguishable. In the mass analysis, the sequence coverage of Rab1 was 27%; thus, it is hard to distinguish between Rab1A from Rab1B (Fig. 5C).

### BALF2 colocalizes with the GTPase Rab1A in the juxtanuclear concave region of Rta reactivated TW01-EBV cells.

**FIG 6:**
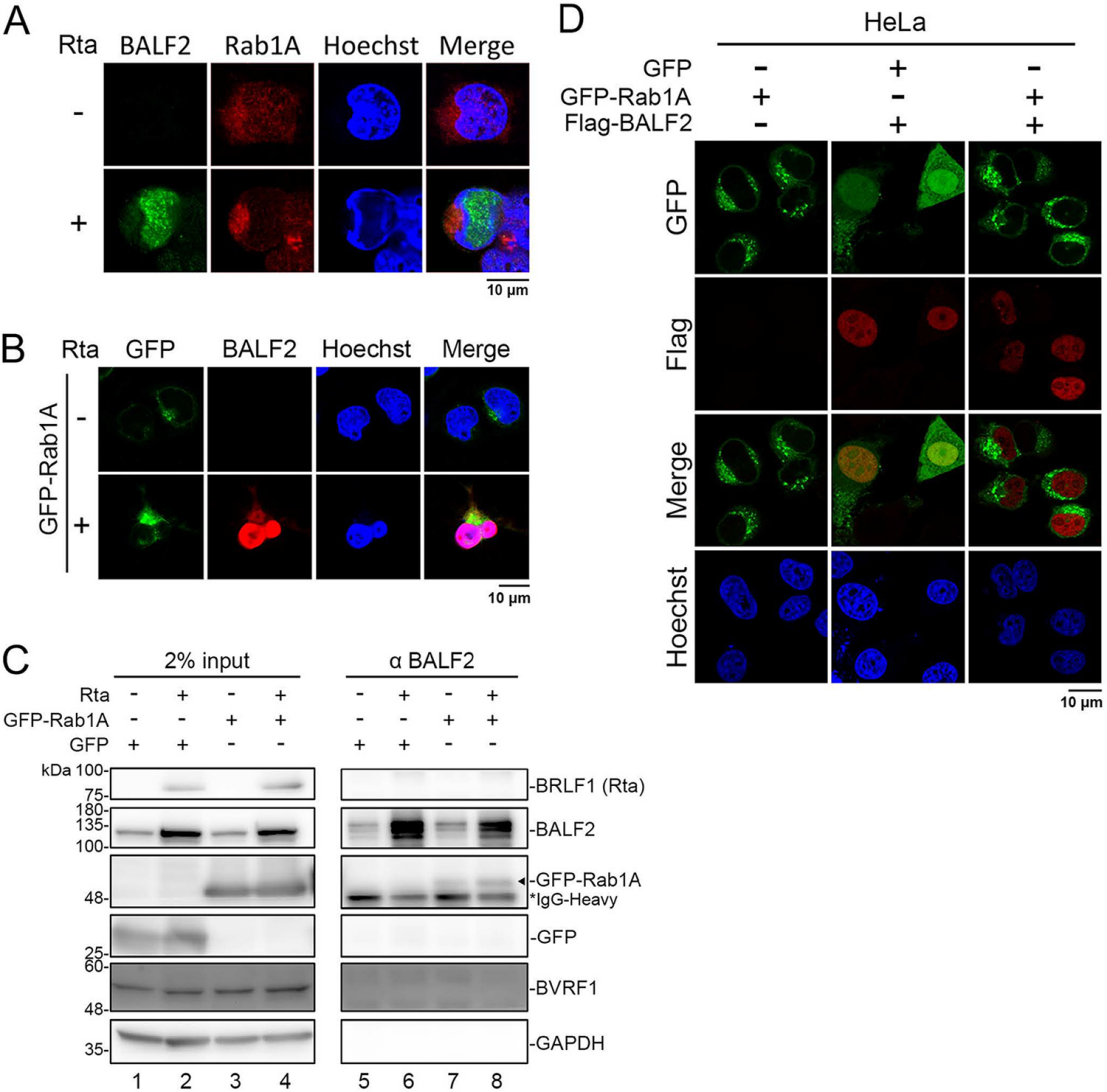
A subset of BALF2 colocalizes with Rab1A in the nuclear concave region in Rta-reactivated TW01-EBV cells. (A) TW01-EBV cells were transfected with pSG5-Rta or pSG5. The distribution of BALF2 and endogenous Rab1A in TW01-EBV cells was detected with mouse anti-BALF2 monoclonal antibody (OT13B) and rabbit anti-Rab1A monoclonal antibody (Cell Signaling, D3X9S) and observed by confocal microscopy at 48 hpt. (B) The distribution of BALF2 and GFP-Rab1A was detected with specific antibodies and observed by confocal microscopy at 48 h post-Rta transfection in TW01-EBV cells. Cells with patterns of BALF2 localized in both the nucleus and viral assembly compartment were 25 out of 30 of BALF2-positive cells. (C) Coimmunoprecipitation of BALF2 and GFP-Rab1A using antibodies against BALF2, GFP, and GST was carried out using TW01-EBVcell lysates harvested at 48 h posttransfection of the Rta-expressing plasmid. Immunoblotting was carried out with BALF2 antibody or GFP antibody. The GFP-Rab1A band is indicated with a black arrowhead. (D) GFP-Rab1A and Flag-BALF2 were coexpressed in HeLa cells. The distribution of Flag-BALF2 and GFP-Rab1A was detected with Flag antibody and observed under confocal microscopy.

In addition to the proteomic analysis, we also noticed that Rab1A was found to be a cellular component in the EBV tegument layer in a previous study (1). Rab1A is known to interact with its effector GM130 in *cis*-Golgi, and our results confirmed that BALF2 and GM130 colocalized in TW01-EBV cells (Fig. 1D). We were prompted to examine the role of Rab1A in the recruitment of BALF2 to the cytoplasmic AC in the juxtanuclear concave region and virion release. Immunostaining and confocal microscopic analysis revealed that endogenous Rab1A is targeted to the juxtanuclear concave region of TW01-EBV cells, where it colocalizes with BALF2 at 48 h post Rta plasmid transfection (Fig. 6A). Joint clustering of GFP and BALF2 at this site became more prominent when the Rta- and GFP-Rab1A-expressing plasmids were cotransfected into TW01-EBV cells in at least 25 out of 30 cells. (Fig. 6B). The interaction between BALF2 and GFP-Rab1A was confirmed by coimmunoprecipitation with the anti-BALF2 antibody OT13B (Fig. 6C, lane 8). To examine whether the colocalization of BALF2 and Rab1A at the viral AC requires other viral factors, we coexpressed Flag-BALF2 and GFP-Rab1A in HeLa cells and could not observe either the juxtanuclear clustering or the colocalization of these two proteins by immunostaining (Fig. 6D), suggesting that other viral factor(s) may cause the clustering of Rab1A in the juxtanuclear region and help the targeting of BALF2 to the viral AC. Unlike the reciprocal coimmunoprecipitation of BALF2 and HA-BVRF1 in the 293T-transiently transfected lysates (Fig. 4E), BVRF1 was not pulled down by BALF2 in Rta and GFP-Rab1A cotransfected TW01-EBV cell lysates (Fig. 6C), possibly because the protein-protein interaction network may be more complex in virus-replicating cells and could not be detected in the coimmunoprecipitation experiment all the time. Considering the data in Fig. 4, we postulate that a portion of BALF2 is translocated into the AC through the interaction with BVRF1 on the assembled nucleocapsids and transported into the cytoplasm in a nuclear egress complex (NEC)-dependent manner. The membrane-associated Rab1A then helps to wrap tegument components with the nucleocapsid for final envelopment.

### Rab1A GTPase activity is essential for BALF2 targeting to the AC.

**FIG 7:**
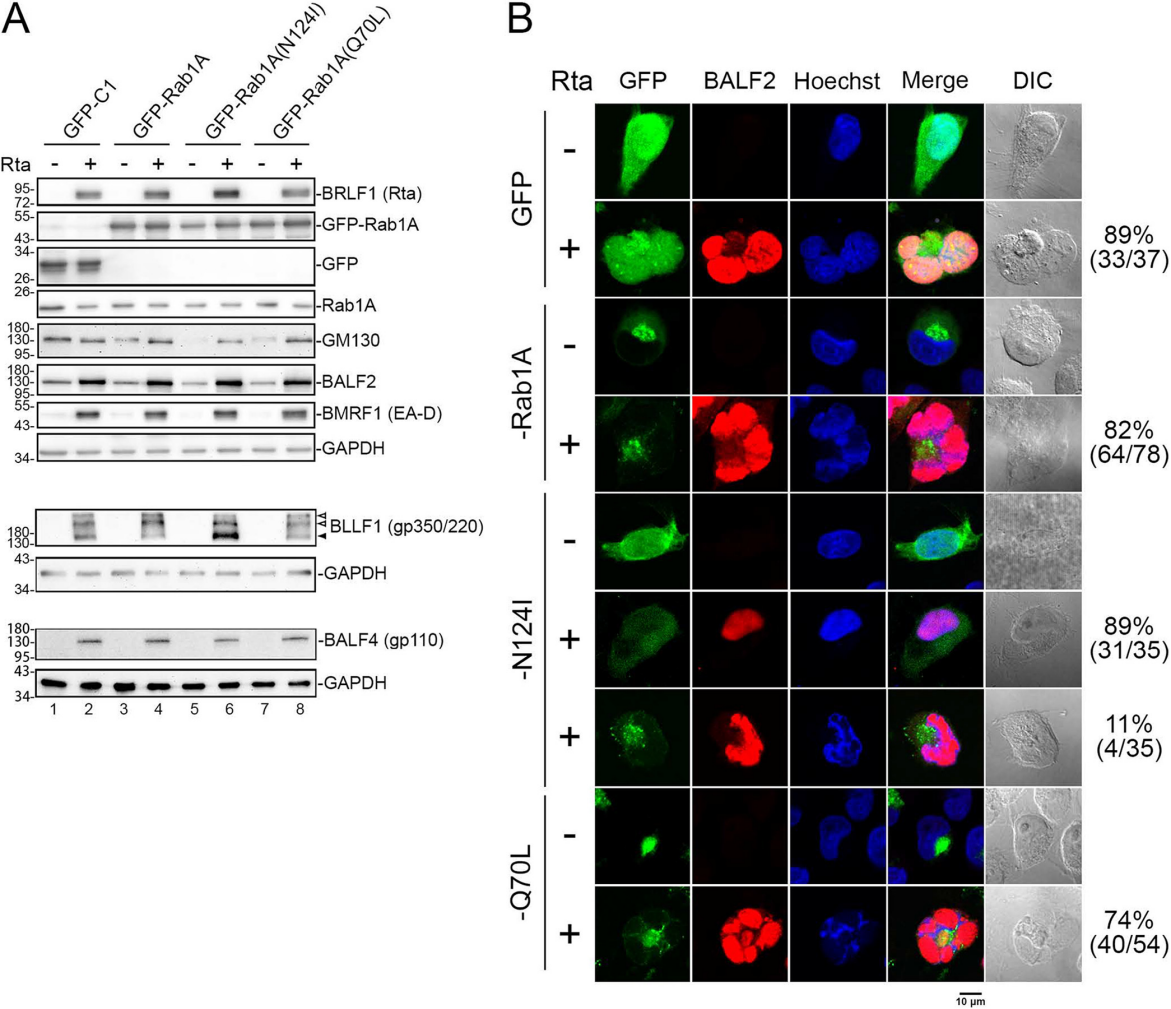
Expression of dominant-negative GFP-Rab1A(N124I) diminishes the clustering of BALF2 to the viral assembly compartment. (A) TW01-EBV cells were cotransfected with pSG5-Rta or pSG5 plus GFP-C1, GFP-Rab1A, GFP-Rab1A N124I, or GFP-Rab1A Q70L. The cell lysates were harvested at 48 hpt. The expression levels of viral proteins BALF2, BMRF1 (EA-D), BLLF1 (gp350/220), and BALF4 (gp110) and cellular proteins GM130 and Rab1A were detected with BRLF1 antibody, BALF2 antibody, BMRF1 antibody, gp350/220 antibody, gp110 antibody, GM130 antibody, and Rab1A antibody in immunoblotting. Transfected GFP, GFP-Rab1A, and BRLF1 (Rta) were also detected in the blotting. GAPDH served as the loading control. Proteins in the top panel were displayed in regular 10% SDS-PAGE, whereas gp350/220 and gp110 were detected in immunoblotting after nonreducing 8% SDS-PAGE. Open arrowheads indicate mature gp350 or gp220, while the solid arrowhead indicates the precursor of gp350/220. (B) The distribution of BALF2 with different GFP-Rab1A constructs was observed by immunostaining and confocal microscopy at 48 h post cotransfection of Rta and GFP, GFP-Rab1A, GFP-Rab1A(N124I), or GFP-Rab1A(Q70L). The percentages listed next to each group were calculated to indicate cells expressing BALF2 localized to the viral assembly compartment or not over GFP^+^ GM130^+^ cells.

To investigate the contribution of Rab1A GTPase activity on the appropriate formation of the viral AC and the targeting of BALF2 to this compartment, we cotransfected Rta-expressing plasmid with GFP-C1, GFP-Rab1A, GFP-Rab1A(N124I) (a dominant-negative Rab1A mutant lacking GTPase activity), or GFP-Rab1A(Q70L) (a constitutively active mutant of Rab1A) into TW01-EBV cells. Western blot analysis indicated that the expression levels of viral proteins, including BALF2, BMRF1 (EA-D), and viral glycoprotein protein BALF4 (gp110) are similar in all settings (Fig. 7A). Because the maturation of EBV needs the final envelopment of nucleocapsids by viral glycoprotein-containing membranes, we also examined the expression pattern of gp350/220 (Fig. 7A, bottom panel). Typically, gp350/220 exhibits three bands in immunoblotting at 350 kDa, 220 kDa, and 165 kDa, which represent gp350, gp220, and their shared precursor, respectively (28). Indeed, we found that the EBV glycoprotein gp350/220 (BLLF1) in the GFP-C1 vector-transfected cells displayed this typical pattern of three bands, i.e., gp350 (top open arrowhead), gp220 (bottom open arrowhead), and their common precursor (black arrowhead). The pattern of gp350/220 in cells transfected with GFP-Rab1A or GFP-Rab1A(Q70L) exhibited enhancement of the upper bands (Fig. 7A, lane 8), whereas the gp350/220 band of the lowest molecular weight was enhanced in GFP-Rab1A(N124I)-transfected cells (Fig. 7A, lane 6), suggesting that Rab1A is also involved in the fully glycosylation of gp350/220.

Next, we examined the subcellular distribution patterns of BALF2 in Rta-reactivated TW01-EBV cells in the presence of wild-type, dominant-negative, or constitutive active forms of GFP-fused Rab1A at 48 h post-Rta transfection. We found that BALF2 localized to the juxtanuclear concave region in most cells transfected with GFP-C1 vector (89%), GFP-Rab1A (82%), or constitutively active GFP-Rab1A(Q70L) (74%). Only a small subset of GFP-Rab1A(N124I)-transfected cells (11%) displayed the distribution of BALF2 to this area, while the remaining 89% of cells displayed more diffuse GFP-Rab1A signals with no BALF2 clustering to the nuclear concave region (Fig. 7B). The data here suggest that Rab1A activity promotes the recruitment of BALF2 to the AC.

### Rab1A GTPase activity is required for the recruitment of fragmented *cis*-Golgi elements to the viral AC.

**FIG 8:**
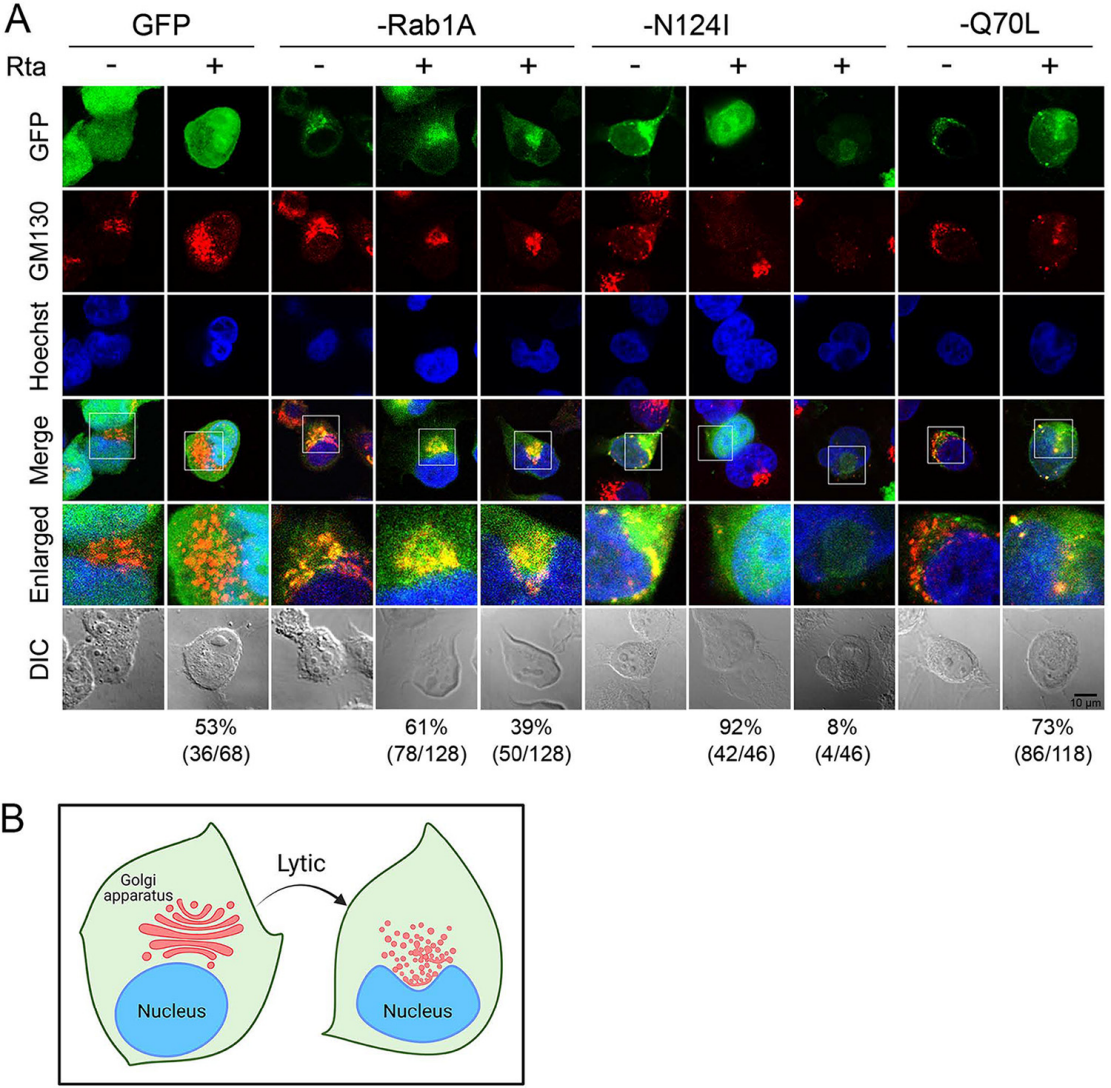
Expression of GFP-Rab1(N124I) disrupts the Golgi structure and attenuates the recruitment of fragmented *cis*-Golgi to the viral assembly compartment during EBV reactivation. (A) The distribution of *cis*-Golgi marker GM130 under the same setting as in Fig. 7B. The *cis*-Golgi compartments in wild-type and mutant GFP-Rab1A transfected TW01-EBV cells are enlarged. In wild-type GFP-Rab1A transfected cells, 61% showed perinuclear Golgi staining after Rta transfection, while 39% displayed disrupted Golgi structure distributed to the nucleus concave region after lytic replication. The percentages listed under each group were calculated as cells expressing GM130 in the viral assembly compartment or not over GFP positive cells. (B) A diagram indicating that EBV replication induces the fragmented Golgi apparatus clustered at the juxtanuclear region and colocalized with wild-type GFP-Rab1A is illustrated. In GFP-Rab1A (N124I) and Rta-transfected cells, GM130 staining indicates that the Golgi stack was disrupted and became diffuse after Rta transfection in 92% of cells. Weak staining signals of GFP and GM130 colocalized in the nuclear concave region were observed in a few cells (8%). In GFP-Rab1A(Q70L)-transfected cells, 73% of cells displayed more significant Golgi fragmentation after Rta transfection. The percentages listed under each group were calculated as cells expressing GM130 in the viral assembly compartment over GFP-positive cells.

It has been reported that EBV acquires the final envelope in Akata cells by budding at intracellular compartments containing Golgi markers (18). Because the complete glycosylation of gp350/220 occurs at the Golgi apparatus, here, we further explored whether Rab1A is involved in BALF2 and gp350/220 targeting to the Golgi-related viral AC. The distributions of the *cis*-Golgi protein GM130 and gp350/220 in wild-type or mutant Rab1A-expressing cells were then examined. Before EBV reactivation, the GM130 staining in GFP vector- and GFP-Rab1A-expressing cells manifested reticular patterns, while fragmentation of the *cis*-Golgi membranes was observed in dominant-negative GFP-Rab1A(N124I). The enlargement of Golgi cisternae was observed in cell-expressing constitutive active GFP-Rab1A(Q70L) without Rta transfection (Fig. 8A), suggesting that proper Rab1 activity is required for Golgi homeostasis (29). In the Rta-expressing cells, the Golgi apparatus is highly fragmented in the juxtanuclear region in 53% of GFP-transfected cells, in 39% of the GFP-Rab1A, and in 73% of the GFP-Rab1A(Q70L)-transfected cells (as illustrated in Fig. 8B). However, in dominant-negative GFP-Rab1A(N124I)-expressing cells, GM130 staining was barely detectable after Rta transfection (92% of cells), even if the juxtanuclear clustering of weak GFP-Rab1A signal was observed (8% of the cells), suggesting that Rta induction in the presence of GFP-Rab1A(N124I) caused disappearance or degradation of GM130. In cells expressing constitutively active GFP-Rab1A(Q70L), GM130 localized to the juxtanuclear concave region and displayed a dispersed pattern upon EBV reactivation (Fig. 8A), suggesting that excessive Rab1 activity enhances virus replication-mediated disruption of the Golgi apparatus.

### Rab1A GTPase activity is required for proper glycosylation of gp350/220.

**FIG 9:**
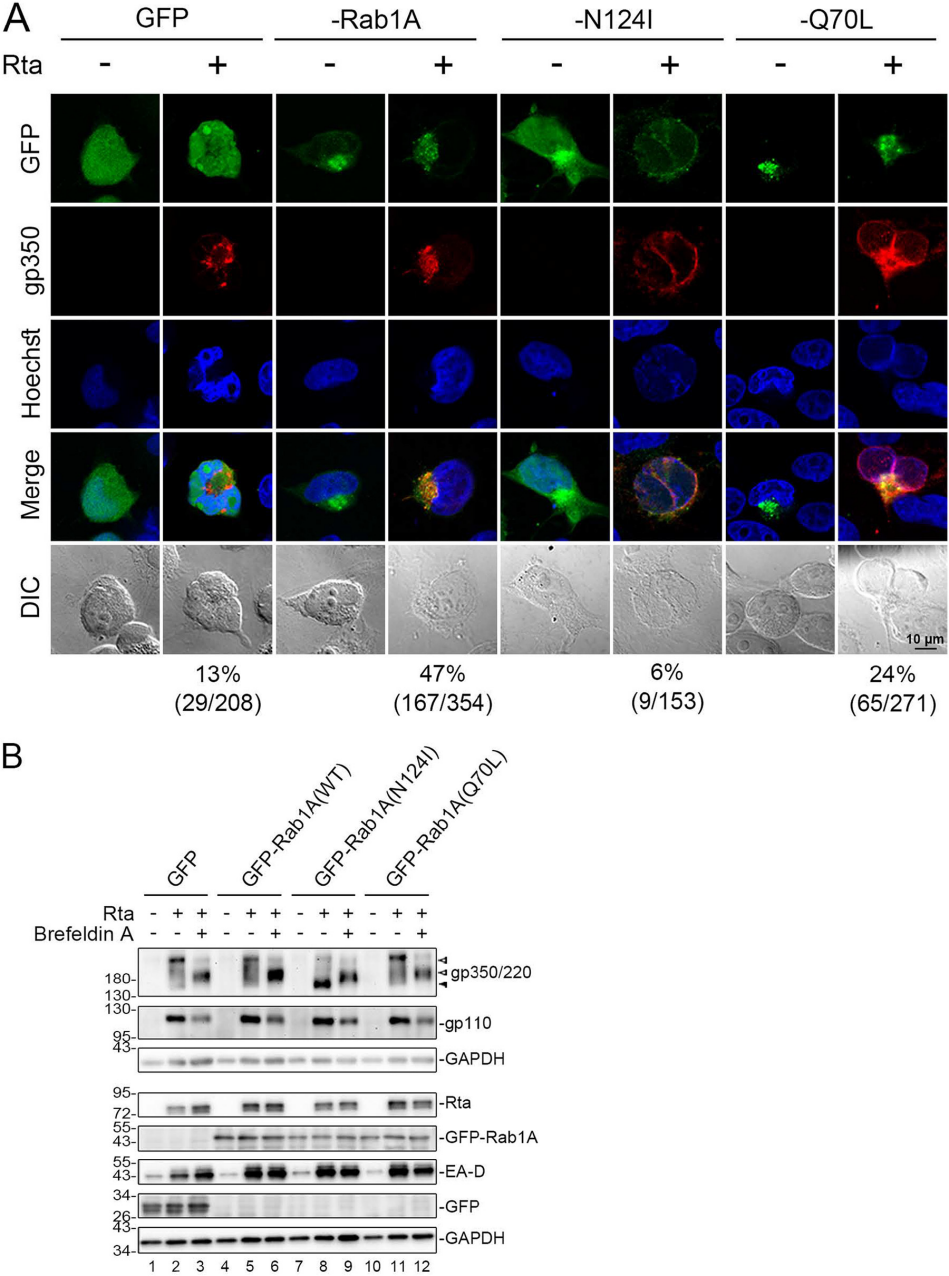
Expression of GFP-Rab1(N124I) interferes with glycoprotein gp350/220 maturation and targeting to the viral assembly compartment. (A) The distribution of EBV glycoprotein gp350/220 under the same setting as in Fig. 7B. The percentages listed under each group were calculated as cells expressing a shown pattern of gp350/220 over GFP^+^ cells. (B) TW01-EBV cells were cotransfected with plasmids expressing Rta and GFP-C1, GFP-Rab1A, GFP-N124I, or GFP-Q70L. TW01-EBV cells transfected with the Rta-expressing plasmid or vector were treated with DMSO or brefeldin A (1 μg/mL) at 24 hpt, and cell lysates were harvested after another 24 h of incubation. Expression of BLLF1 (gp350/220) and BALF4 (gp110) were analyzed by nonreducing 8% SDS-PAGE followed by immunoblotting at 48 h posttransfection. Hypo-glycosylated gp350/220 (about 165 kDa) is indicated with a black arrowhead, while glycosylated gp350 and gp220 are indicated with open arrowheads. Lanes 3, 6, 9, and 12 were treated with brefeldin A (1 μg/mL) for Golgi disruption at 24 h post-Rta transfection.

Under the same conditions, the localization of the EBV glycoprotein gp350/220 to the viral AC was seen in 13%, 47%, or 20% of the GFP-vector-, GFP-Rab1A-, or GFP-Rab1A(Q70L)-transfected TW01-EBV cells (Fig. 9A). In particular, gp350/220 was only weakly detected in 6% of GFP-Rab1A(N124I)-expressing cells. Immunoblotting analysis of the GFP-Rab1A(N124I)-expressing cells showed the enrichment of the gp350/220 band at around 165 kDa, probably corresponding to the hypo-glycosylated precursor of gp350/220 (Fig. 7A, black arrowhead). To further investigate whether this lowest-molecular-weight 165-kDa band of gp350/220 precursor is indeed an enriched hypo-glycosylated form, we treated the EBV-reactivated TW01-EBV cells with brefeldin A (BFA), which is an inhibitor of the assembly of COPI vesicle coats and may cause the Golgi apparatus to collapse back to the ER (30). We then compared the patterns of gp350/220 in wild-type or mutant GFP-Rab1A-expressing cells. In response to BFA treatment, the immature form of gp350/220 enriched in BFA-treated cells showed slower migration than that of the 165-kDa precursor of gp350/220 (Fig. 9B, lanes 3, 6, 9, and 12). A previous study showed that, over time, retention of glycoproteins in the ER may undergo a low level of O-linked glycosylation but fail to be processed to the mature form (29). We speculated that the band with slower migration could be an immature O-linked glycosylated form of gp350/220; it is similar to the case of pseudorabies virus gp50 in the presence of BFA (31). In the presence of GFP-Rab1A(N124I), the band of gp350/220 is most likely the nonglycosylated form (Fig. 9B, lane 8). Taken together, these results suggest that the dominant-negative Rab1A disrupts the formation of the viral AC in TW01-EBV cells by inhibiting the localization of the viral tegument protein BALF2 and gp350/220 to the juxtanuclear concave Golgi area. In addition, the GTPase activity of Rab1 is required for appropriate glycosylation of gp350/220.

### Expression of dominant-negative Rab1A attenuates EBV virion release and infectivity.

**FIG 10:**
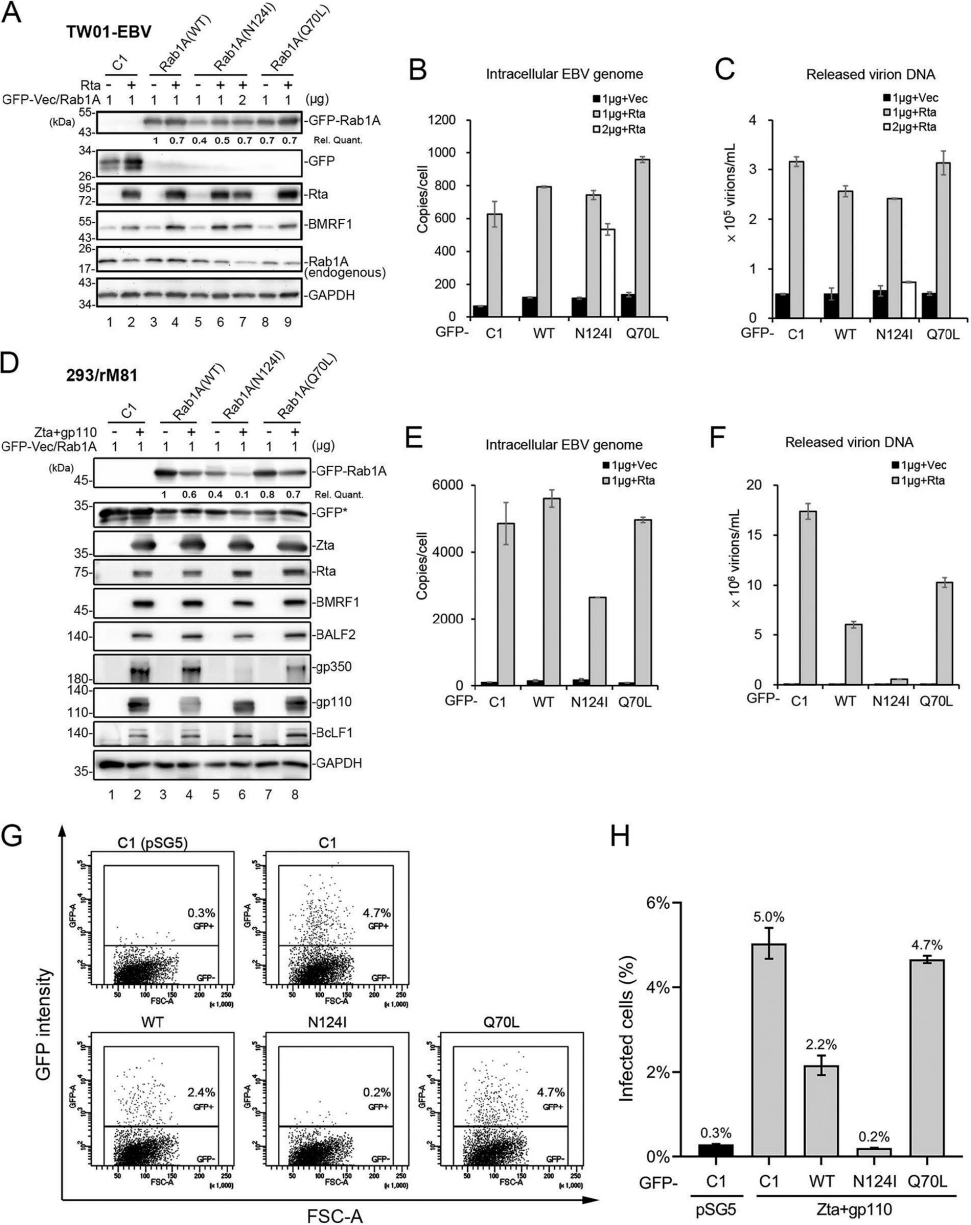
Expression of GFP-Rab1(N124I) in TW01-EBV and 293/rM81 cells attenuates EBV release and infectivity. (A) TW01-EBV cells were transfected with pSG5-Rta or pSG5 plus GFP, GFP-Rab1A, GFP-Rab1A(N124I), or GFP-Rab1A(Q70L) using Lipofectamine 2000. The cell lysates were harvested for immunoblotting at 48 hpt. The expression levels of Rta, GM130, endogenous Rab1A, BALF2, and wild-type, dominant-active, or constitutive GFP-Rab1A in TW01-EBV cells were detected by GFP antibody, BRLF1 antibody, BMRF1 antibody, and Rab1A antibody. Gp110 and gp350/220 were analyzed by 8% SDS-PAGE (bottom panels). (B) The intracellular EBV genomic DNA copy numbers of TW01-EBV cells were analyzed by q-PCR at 48 hpt of Rta and GFP-C1 vector or GFP-Rab1A constructs. (C) The released EBV virion DNA in the medium in the same setting was analyzed by q-PCR at 48 hpt. The representative data from 4 independent experiments are displayed. (D) 293/rM81 cells were cotransfected with Zta and gp110 expression plasmids or pSG5 control plus GFP, GFP-Rab1A, GFP-Rab1A(N124I), or GFP-Rab1A(Q70L) using TransIT-LT1. Cell lysates were harvested for immunoblotting at 72 hpt. The expression levels of Zta, Rta, BMRF1, BALF2, and wild-type, dominant-active, or constitutive GFP-Rab1A in 293/rM81 cells were detected using specific antibodies. For detecting gp350/220, gp110, and BcLF1, lysates were displayed in nonreducing 8% SDS-PAGE. (E) The intracellular EBV genomic DNA copy number of 293/rM81 cells was analyzed by q-PCR at 72 hpt of Zta, gp110, and GFP-C1 vector or GFP-Rab1A constructs. (F) The released EBV virion DNA of the same setting was analyzed with q-PCR at 72 hpt. (G) Culture supernatants from the experiment in panel D were filtered through a 0.45-μm filter, and that same volume of filtered viral supernatant was added to Raji cells and incubated for 72 h in duplicated wells. The percentages of Raji cells expressing GFP were determined through flow cytometry. The results of one experiment are presented. The horizontal axis represents the forward scatter area (FSC-A), and the vertical axis indicates the intensity of GFP fluorescence (GFP-A). The GFP positivity threshold was determined according to the noninduction control (C1-pSG5). Experimental results are presented using the mean with standard deviation (SD) as the bar graph in panel H.

To examine the role of Rab1A in EBV virion maturation and release, we measured the intracellular EBV genomic DNA replication and virion release in TW01-EBV cells by transfecting the Rta-expressing plasmid together with the GFP vector or the various GFP-Rab1A constructs. Immunoblotting revealed that protein levels of GFP-Rab1A(N124I) were less if the same amounts of plasmid DNA were transfected into TW01-EBV cells (Fig. 10A, lanes 5 and 6). Therefore, we transfected 1 or 2 μg of GFP-Rab1A(N124I) and 1 μg of GFP, GFP-Rab1A, or GFP-Rab1A(Q70L) into TW01-EBV cells (Fig. 10A). At similar expression levels, the intracellular EBV genomic DNA copy number in GFP-Rab1A(N124I)-transfected cells was similar to other constructs (Fig. 10B, 2 μg), while virion release from GFP-Rab1A(N124I)-transfected cells was attenuated (Fig. 10C, 2 μg).

In order to demonstrate the dominant-negative effect of Rab1A(N124I) on virion infectivity, 293/rM81, which is a 293-derived line containing a recombinant M81 EBV strain that was cloned with an F-factor and the GFP gene (32), was used for virus production to superinfect Raji cells. To this end, 293/rM81 cells were transfected with Zta and gp110 expression plasmids to induce lytic replication and virion release in the presence of vector or various GFP-Rab1A constructs. Immunoblotting revealed that protein levels of GFP-Rab1A(N124I) were less than the GFP-Rab1A wild type (WT) or Q70L when the same amounts of plasmid DNA were transfected into 293/rM81 cells (Fig. 10D). The protein levels of Zta, Rta, BMRF1, BALF2, gp110, and BcLF1 (VCA) were similar by GFP-Rab1A(N124I) expression. Notably, gp350/220 was significantly reduced in the presence of dominant-negative Rab1A(N124I) in 293/rM81 cells (Fig. 10D, lane 6). In qPCR analysis, intracellular EBV genomic DNA copy numbers of GFP-Rab1A(N124I) cells were about half those of the other groups at 72 h posttransfection (Fig. 10E), while virions released from GFP-Rab1A(N124I)-transfected 293/rM81 cells were significantly attenuated (Fig. 10F). Then equal volumes of virus supernatant from the GFP-Rab1A-transfected groups were used to superinfect Raji cells and cultured for 72 h. GFP-positive Raji cells were analyzed by flow cytometry (Fig. 10G), and the percentage of GFP-positive cells correlated well to the released copy numbers detected by qPCR in Fig. 10H. In the presence of GFP-Rab1A(N124I), there were very few GFP-positive Raji cells in the superinfection, which may be due to the low expression level of gp350/220 (Fig. 10D, lane 6). Overall, Rab1 activity is required for EBV glycoprotein gp350/220 maturation and virus infectivity.

Because Rab1A and Rab1B share 93% amino acid identity, we also tried to knock down Rab1A, Rab1B, or both by a small interfering RNA (siRNA) approach to examine their possible distinctive functions in TW01-EBV cells. Our results indicate that after Rta transfection, the virus release was only significantly diminished in double knockdown cells, but not in single knockout cells, suggesting that there are redundant functions of Rab1A and Rab1B in promoting EBV maturation (data not shown).

## DISCUSSION

### Subcellular distribution of BALF2 is regulated through protein-protein interactions, and Rab1A is essential for AC formation and final envelopment of EBV.

**FIG 11:**
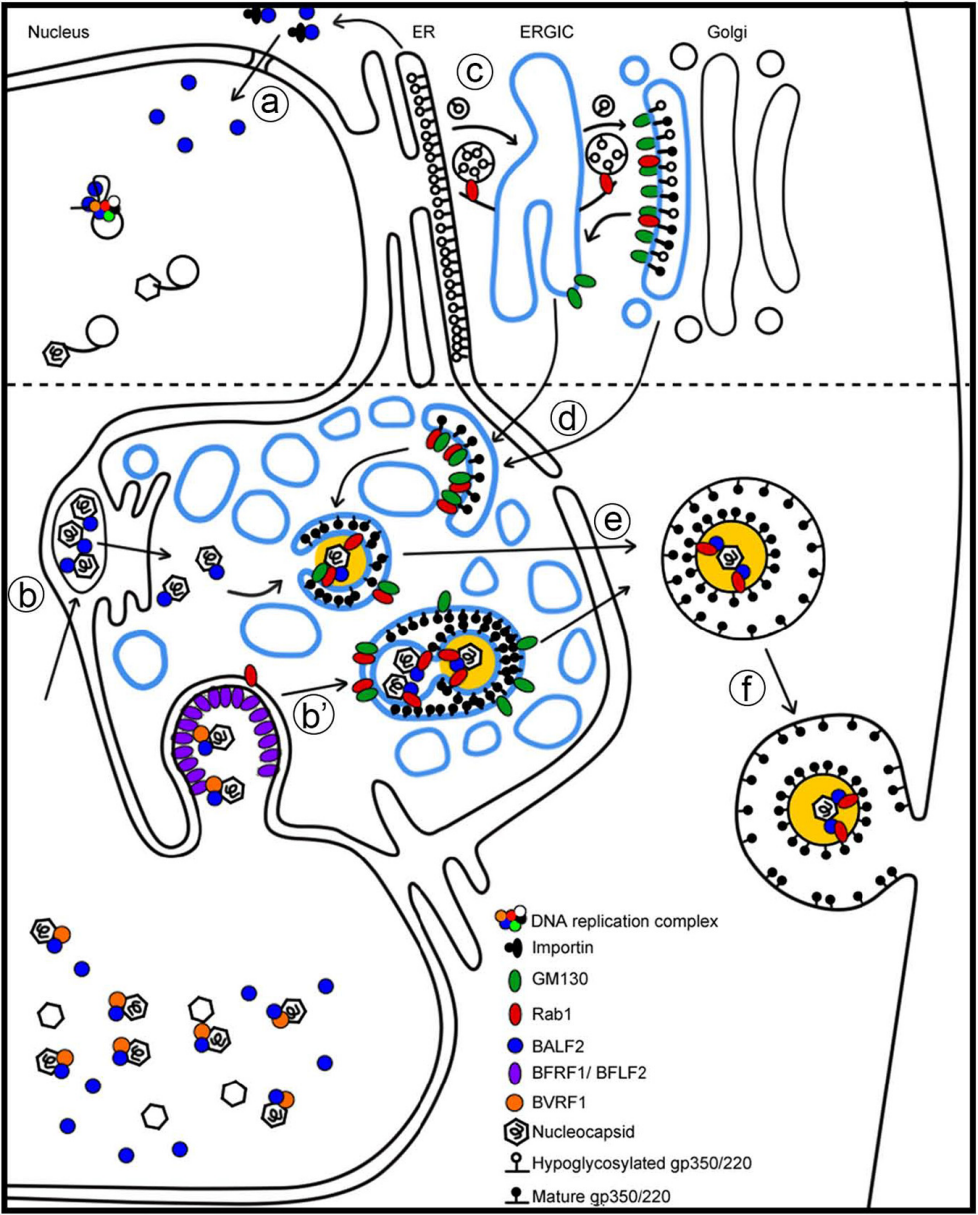
A hypothetical diagram of the BALF2 subcellular distribution during virus replication and the role of Rab1A in the assembly of EBV viral particles. The top part, above the dotted line, indicates early steps of EBV reactivation. (a) BALF2 is translated from the ER and transported into the nucleus through a canonical importin-β-dependent mechanism. (b) At the mid- to late stage of EBV replication, viral DNAs are encapsidated into procapsids, and the nucleocapsids are egressed from the nucleus through either a primary envelopment and deenvelopement process (b) or a nuclear egress complex and ESCRT-dependent mechanisms (b′). (c) After synthesis on the ER membrane, gp350/220 was transported through Rab1-mediated vesicle transport to the *cis*-Golgi apparatus for proper glycosylation. (d) Fragmented Golgi and Golgi-derived vesicles with membrane-associated gp350/220 may target the viral assembly compartment. (e) Rab1 interacts with GM130 and anchors on the *cis*-Golgi membranes and Golgi-derived vesicles. The membrane-associated Rab1 helps the engulfment of nucleocapsids or nucleocapsids containing vesicles through interaction with a BALF2-associated protein-protein interaction. (f) The mature virion with tegument (yellow regions) may be released through exocytosis.

A hypothetic model of nuclear targeting of BALF2 and the contribution of Rab1A in EBV assembly compartment formation and virion release is schematically summarized together with the study of HCMV AC (22) and some of our unpublished data (Y.C. Dai et al., manuscript in preparation) in Fig. 11. At the early phase of EBV reactivation, BALF2 is imported into the nucleus through the canonical nuclear targeting mechanism, where it functions in viral DNA replication (step a). After EBV genome replication, newly synthesized DNA is encapsidated into procapsids, after which the nucleocapsids are exported to the cytoplasm through nuclear egress complexes with an ESCRT-dependent pathway (step b′) or an alternative pathway involving envelopment and deenvelopment (step b). We propose that the intranuclear BALF2 may associate with the nucleocapsids, possibly via BVRF1 interaction, to be transported into the cytoplasm in a BFRF1/BFLF2 nuclear egress complex (NEC)-dependent manner. Simultaneously, newly synthesized precursors of gp350/220 are transported from ER to *cis*-Golgi via the ER-Golgi intermediate compartment (ERGIC) by Rab1-regulated trafficking, undergoing further glycosylation in the Golgi apparatus (step c) or in the ERGIC-derived vesicles (colored in blue) enriched in the juxtanuclear concave area (step d). Subsequently, the nucleocapsids arriving in the cytoplasm undergo final envelopment, which may require the interaction between the nucleocapsid-associated BALF2, other viral and cellular tegument proteins, and Rab1-associated ERGIC-derived membranes (step e). At this step, the fragmented Golgi-derived membranous structure forms a large viral assembly compartment with various viral and cellular factors. The mature virions are then released to the extracellular space via an exocytosis mechanism (step f). Thus, both BALF2 and Rab1 are incorporated into the tegument layer of mature virions. The reorganized ER structure surrounding the Golgi apparatus was drawn according to Fig. 8 and confocal analysis in our other study for the organization of assembly compartments with different organelle markers (Y.C. Dai et al., manuscript in preparation).

The replication process of herpesviruses not only involves a cascade of regulation of viral gene expression for viral DNA replication and synthesis of structural proteins, but also needs to hijack and modify various cell machineries to overcome the spatial and physical barriers of the cell. During lytic replication, components of the EBV-encoded DNA replication complex need to be transported into the nucleus for viral genome replication. Interestingly, several viral proteins building this replication complex do not contain a nuclear localization signal. For example, the EBV-encoded DNA polymerase BALF5 and viral uracil DNA glycosylase BKRF3 need to associate with the DNA polymerase processivity factor BMRF1 to be transported into the nucleus (10, 33), whereas the primase-associated proteins, including BSLF1, BBLF2/3, and BBLF4, are guided into the nucleus by BGLF4 kinase via a mechanism that remains unclear (11). Our results here revealed that the nuclear import of BALF2 is mediated by its C-terminal NLS and the canonic importin-dependent nuclear localization pathway. Fusion of 29 C-terminal amino acids of BALF2 can also target LacZ into the nucleus (Fig. 2 and 3).

We were curious about the subcellular distribution BALF2 at different stages of EBV lytic replication and how BALF2 is incorporated into virions. We also confirm that some BALF2-associated proteins, which were identified in a yeast two-hybrid screening, may contribute to the transport of BALF2 to the AC and the mid-stage of virus replication. We suggest that the interaction with capsid-associated tegument protein BVRF1 may lead to the nucleocytoplasmic transport of nucleocapsid-associated BALF2 through the NEC BFRF1/BFLF2-dependent remodeling of nuclear membrane (Fig. 4A). The subsequent EBV BGLF4 kinase-mediated nuclear lamin A/C phosphorylation and the partial disassembly of nuclear lamina may then promote the nuclear egress process. A similar protein-protein interaction was also observed in HCMV nuclear egress. In coimmunoprecipitation and immunogold staining for electron microscopy (EM) observation, the HCMV NEC component pUL53 (the homolog of EBV BFLF2) was found to be directly associated with the viral kinase UL97 and the association of both nuclear capsids and NEC proteins at nuclear lamina budding sites (34). Here, we demonstrated that BVRF1 coimmunoprecipitated BFRF1 and BGLF4 (Fig. 4C), and the distribution of BALF2 and BVRF1 in the juxtanuclear AC of Rta reactivated TW01-EBV cells (Fig. 4D and E). This indicates that a complex viral protein-protein interacting network may ensure the successful assembly of viral tegument components. However, some of these interactions may be transient for subsequent interactions, or the large protein-interacting complex may prevent the coimmunoprecipitation of individual interacting protein pairs.

In the search for cellular proteins that can facilitate the incorporation of BALF2 into the tegument, we found an interesting group of proteins that are involved in COPI-dependent Golgi-to-ER retrograde traffic. Because GTPase Rab1A participates in ER-Golgi trafficking and maintenance of the Golgi structure, we explored the contribution of Rab1 in viral morphogenesis further. Our data demonstrated that Rab1 is required for BALF2 targeting to the AC, the maturation of the glycoprotein gp350/220, as well as the release of mature virions from TW01-EBV cells. As Rab1A is a specific marker of the ER-Golgi intermediate compartment (ERGIC) (35), our results suggest that the AC of EBV could be an ERGIC-derived structure. In addition, the AC also contains GM130, which is generally considered a *cis*-Golgi marker but is also present in the ERGIC (35, 36). Notably, the expression of dominant-negative Rab1A interfered with the targeting of viral proteins to the juxtanuclear concave region where the AC resides, as indicated by localization of the EBV tegument protein BALF2 and glycoprotein gp350/220. In addition, the expression of dominant-negative Rab1A may also reduce the transport of mature viral particles and additional releasing processes (Fig. 9A) and the reduction of virion release (Fig. 10C and F). We propose that the viral assembly compartments of EBV and HCMV may be structurally similar and both be derived from the ERGIC membranes surrounding the microtubule-organizing center (MTOC)/centrosome (36). The nucleocapsid-associated BALF2 may recruit Rab1-bound ERGIC membranes directly or indirectly and subsequently help the closure of the EBV envelope and the incorporation of BALF2 and Rab1 into the tegument layer (Fig. 11). It remains unclear whether the virions containing BALF2 and Rab1 are the residual components from intracellular assembly of mature virions or have specific functions for the next round of infection. As a master regulator of membrane transport and tethering during ER-Golgi trafficking and autophagy (37), Rab1 has been shown to be involved in the maturation and secretion of several viruses, such as HSV-1 (38, 39). Zenner and coworkers investigated 37 TBC (Tre-2, Bub2 and Cdc16) domain-containing Rab GAPs to screen for their abilities to inhibit HSV-1 replication. Among these candidates, overexpression of TBC1D20 and RN-tre caused a reduction of HSV-1 virus titer, and therefore their targets, Rab1 and Rab43, were picked up for siRNA knockdown experiments and glycosylation pattern analysis of the viral glycoproteins. They demonstrated that Rab1A depletion reduces the glycosylation of glycoprotein D (gD), but not of gB or gH, while Rab1B depletion alone exerted minor effects on the glycosylation of these three HSV-1 glycoproteins. However, depletion of both Rab1A and Rab1B abolished the glycosylation of all of them (gD, gB, and gH) (39). In addition, Rab1 has been demonstrated to play important roles in the replication of other enveloped viruses, including hepatitis C virus (HCV) (40), HIV-1 (41), vaccinia virus (42), and classical swine fever virus (43). Interestingly, Rab1 mediates the glycosylation of HIV-1 gp41, but not gp160, indicating that not all viral glycoproteins depend on Rab1 for their maturation (41). Taken together, Rab1A and Rab1B possess high similarity and may have partial redundant effects on the glycosylation of viral glycoproteins. Our results revealed that the dominant-negative Rab1A abolished the maturation of EBV glycoprotein gp350/220 and reduced mature virion release and the infection of Raji cells.

Notably, several Rab proteins besides Rab1A were detected in our mass spectrum analysis of BALF2-interacting proteins, including Rab7A, Rab13, Rab14, Rab18, and Rab21. This suggests that BALF2 behaves as a component of viral DNA replication complex in the nucleus, as well as an adaptor protein in the cytoplasm for hijacking Rab-mediated intracellular trafficking processes as a tegument protein. These Rab proteins are known to function in post-Golgi or Golgi bypass trafficking, such as autophagosome-lysosome fusion (Rab7A) (44), Golgi-endosome trafficking (Rab14) (45), endocytic recycling (Rab13 and Rab21) (46, 47), transport to specific plasma membrane domains (Rab13) (48), or ER-lipid droplet communication (Rab18) (49). However, only Rab1A was identified as a component of the mature EBV virion (1), suggesting its role in tegumentation and final EBV envelopment. It is worth mentioning that Rab7A-mediated autophagosome-lysosome fusion is blocked during the EBV lytic cycle to promote virus replication (50) and virion production (51). One possibility is that early autophagosome-like membrane structures may be used as precursors for the viral envelope and that late autophagosome-lysosome fusion is prevented by viral factors(s).

In summary, our study reveals how the viral single-stranded DNA binding protein BALF2 is targeted to different subcellular compartments at different stages of virus lytic replication. The tegumentation and final envelope of EBV required the coordination of glycoprotein-associated membrane structures in a Rab1A-dependent manner. Thus, studies of BALF2 and other Rab proteins may lead to further insights regarding viral-host cell interplay and the role of secretory and endocytic pathways in the EBV life cycle.

## MATERIALS AND METHODS

### Cell culture and treatment.

EBV-positive TW01-EBV cells, which were derived from NTUTW01 (abbreviated as TW01) (52) converted with recombinant Akata strain EBV (53), were used. TW01-EBV cells were cultured in RPMI 1640 medium (HyClone) supplemented with 8% fetal bovine serum (GC13475273, SH30084.03, HyClone), penicillin (100 U/mL), streptomycin (100 μg/mL), and 2 mM l-glutamine at 37°C with 5% CO_2_. HeLa cells and 293T cells were cultured in Dulbecco’s modified Eagle’s medium (DMEM; HyClone) supplemented with 10% fetal bovine serum, penicillin (100 U/mL), streptomycin (100 μg/mL), and 2 mM l-glutamine at 37°C with 5% CO_2_. EBV-positive B95-8 and Raji cells were cultured in RPMI 1640 medium supplemented with 10% fetal bovine serum, penicillin (100 U/mL), streptomycin (100 μg/mL), and 2 mM l-glutamine at 37°C with 5% CO_2_. EBV reactivation in TW01-EBV cells was induced by transfection of BRLF1 (Rta) expression plasmid pSG5-Rta or by treatment of 1.25 μM trichostatin A (TSA; T8552, Sigma). Brefeldin A (B7651, Sigma) was used to treat Rta-transfected TW01-EBV cells at a concentration of 1 μg/mL for Golgi disruption at 24 h post-Rta transfection. 293/rM81 cells were obtained from H. J. Delecluse (German Cancer Research Center (DKFZ), Heidelberg, Germany) (32) and cultured in RPMI 1640 medium (HyClone) supplemented with 10% fetal bovine serum, hygromycin (100 μg/mL), penicillin (100U/mL), streptomycin (100 μg/mL), and 2 mM l-glutamine at 37°C with 5% CO_2_.

### Plasmids.

pSG5-BALF2 (pYC5) was derived from pRTS12, M-ABA strain BALF2 driven by SV40 promoter (a gift from Diane Hayward, Department of Pharmacology and Molecular Sciences, Johns Hopkins School of Medicine, USA) (54), by reverse mutagenesis with primers LMRC681, LMRC682, LMRC683, and LMRC684 to obtain the B95-8 strain BALF2 coding sequence with 7 synonymous substitution sites. For BALF2 deletion mutants, the original constructs were generated in pSG5 backbone, and later mutants were cloned into pCMV-Flag for equal detection sensitivity of mutant proteins. Primers LMRC707 and LMRC711 for pSG5-BALF2(Δ1100-1128) (pYC6) were used, and the PCR products were digested with EcoRI and BamHI and cloned into EcoRI and BamHI sites of pSG5. pCMV-Flag-BALF2 (pYC11) was derived from pYC5 by cloning the PCR product with primers LMRC707 and LMRC758 into EcoRI and HindIII sites of pCMV-tag2B. pCMV-Flag-BALF2(Δ1100-1128) (pYR1) was derived from pYC6 using LMRC707 and LMRC1111. pCMV-Flag-BALF2(NLS5A) (pYCS5) was derived from pYC11 using single primer-base mutagenesis using primer LMRC914 to mutate BALF2 amino acids 1113 to 1117 from RRKRR into AAAAA. To confirm the ability of BALF2 C-terminal amino acids 1100 to 1128 (BALF2-C29) for nuclear localization, BALF2-C29 was fused with YFP-LacZ to generate pEYFP-LacZ-C29 (pYYC302) plasmid. Briefly, BALF2-C29 cDNA fragment was amplified with primers LMRC1140 and LMRC1141, in the flanking of 5′-NheI and 3′-HindIII restriction enzyme cutting sites. The NLS positive-control pEYFP-LacZ-NLS, containing the SV40 NLS, (kindly provided by Mitsuhiro Kawata, Kyoto Prefectural University of Medicine, Japan) was linearized by double digestion with SpeI and HindIII to remove the NLS sequence. The restriction enzyme-digested cDNA insert was purified and ligated in-frame with the C terminus of YFP-LacZ in the linearized vector. For NLS negative-control pEYFP-LacZ-MYC (pYYC301) construction, the NLS (PKKKRKVEAY) was replaced by a synthesized SpeI-XhoI-HindII (LEEAY) adaptor (forward-oligonucleotide LMRC1143, reverse-oligonucleotide LMRC1144) and in-frame with C-terminal Myc tag.

GFP-Rab1A was described previously (55). GFP-C1 is a GFP-expressing plasmid (GenBank accession no. U55763; Clontech, Mountain View, CA). Dominant-negative Rab1A defective in guanine nucleotide binding was constructed based on the backbone of GFP-Rab1A, with an asparagine to isoleucine substitution at amino acid 124 (N124I, nucleotides AAC to ATC). Constitutively active Rab1A, defective in GTP hydrolysis, contains a glutamine to leucine substitution at amino acid 70 (Q70L, nucleotides CAA to CTG). GFP-Rab1A(N124I) and GFP-Rab1A(Q70L) were constructed using single primer-based mutagenesis with primer LMRC1069 (according to Nevo-Yassaf’s design) (56) or primer LMRC1070 (according to Ishida’s design) (57) and GFP-Rab1A as the template. The sequence of each primer is listed in Table 1. For induction of rM81 virus production, p509 (BZLF1 expression plasmid, derived from the EBV B95.8 strain driven by cytomegalovirus [CMV] promoter) and p2670 (BALF4 expression plasmid, which encodes the gp110 glycoprotein, both from H. J. Delecluse, Germany), were used.

**TABLE 1:** List of primers used in this research

Primer	Sequence
LMRC681	5′-gtggccaaggtggcccccctcaaggagttccca-3′
LMRC682	5′-ggcgaggcatgcgcctcgctgactagggacg-3′
LMRC683	5′-caactttatcagcgtggccgagccggtca-3′
LMRC684	5′-aagcgccgtctggccaccgttctccccggac-3′
LMRC713	5′-ccagaacagcttcatctcggtccctgtcccca-3′
LMRC707	5′-ggaattcatgcagggtgcacagactagcg-3′
LMRC711	5′-cggatccctacgaggcctgcgacgc-3′
LMRC758	5′-cccaagcttctagacctcgagtccggggaga-3′
LMRC914	5′-gcagggctccgggggcgcagccgcggccgccctggccaccgttctcc-3′
LMRC1069	5′-gtcagatcgcatttgatccctaccaacaact-3′
LMRC1070	5′-gtgattgttcgaaatctttccaggcctgctgtgtcccatatt-3′
LMRC1111	5′-ccaagctttaataagatctggatcc-3′
LMRC1140	5′-tgggctagcgccgggctgctgctgggtg-3′
LMRC1141	5′-cgtaagcttcgacctcgagtccggggag-3′
LMRC1143	5′-ctagtggatctctcgagga-3′
LMRC1144	5′-agcttcctcgagagatcca-3′

### Antibodies.

Primary antibodies used in protein detection, including Western blotting (WB) and immunofluorescence assay (IF), included BALF2 (OT13B; 1:80,000 in bovine serum albumin [BSA] for WB and 1:800 in BSA for IF) (58), Rta (37-1H10; 1:1,000 in skim milk for WB) (59), GFP (GTX113617, GeneTex; 1:1,000 in skim milk for WB), gp350/220 (72A1, ATCC, Manassas, VA; 1:5 in BSA for WB and undiluted for IF), BMRF1 (88A9; 1:100 in skim milk for WB) (60), gp110 (L2) (61), BGLF4 (2224, 1:100 in BSA for IF; 2616, 1:1,000 for WB), and BVRF1 mouse antiserum (1:200 in BSA for WB). The anti-BcLF1 rabbit antiserum was provided by Yasushi Kawaguchi (The Institute of Medical Science, the University of Tokyo). For cellular proteins, GM130 (610822, BD; 1:1,000 in skim milk for WB and 1:100 in BSA for IF), Rab1A (GTX101454, GeneTex; 1:1,000 in skim milk for WB and D3X9S, Cell Signaling; 1:800 for IF), Rab1B (GTX32825, GeneTex; 1:1,000 in skim milk for WB), Flag-M2 (F3165, Sigma; 1:1,000 in skim milk for WB and 1:100 in BSA for IF), HA (16B12, Covance; 1:2,000 in skim milk for WB), and GAPDH (Biodesign; 1:80,000 in skim milk for WB) were used. The secondary antibodies used in protein detection, including Western blotting and immunofluorescence assay, are listed as follows: horseradish peroxidase (HRP)-conjugated goat anti-mouse IgG (115-035-146, Jackson ImmunoResearch; 1:10,000 for WB), HRP-conjugated goat anti-rabbit IgG (AP132P, Millipore; 1:10,000 for WB), fluorescein isothiocyanate (FITC)-conjugated goat anti-mouse IgG antibody (115-095-003, Jackson ImmunoResearch; 1:100 in phosphate-buffered saline [PBS] for IF), Rhodamine-conjugated goat anti-mouse IgG (115-295-146, Jackson ImmunoResearch; 1:100 in PBS for IF), and rhodamine-conjugated goat anti-rabbit IgG (111-295-144, Jackson ImmunoResearch; 1:100 in PBS for IF). The HRP-conjugated secondary antibodies were diluted in TBST buffer (100 mM Tris [pH 7.4], 9% [wt/vol] NaCl, and 2% [vol/vol] Tween 20) for Western blotting.

### Preparation of antiserum against BVRF1.

A single colony of pET30a-His-BVRF1-transformed Escherichia coli BL21(DE3) (a gift from Hsiu-Ming Shih, Academia Sinica, Taiwan) was cultured in LB broth containing 50 μg/mL kanamycin and 1% glucose overnight at 37°C and subsequently expanded into fresh medium according the protocol suggested by the Novagen pET expression manual. In brief, the culture was treated with IPTG (isopropyl-β-D-thiogalactopyranoside; 0.1 mM) and incubated at 24°C for 3 h, and the bacteria were pelleted by centrifugation and resolved in bacterial lysis buffer (20 mM imidazole, 50 mM NaH_2_PO_4_, 300 mM NaCl [pH 8.0]) with lysozyme (1 mg/mL) on ice for 30 min subsequent to sonication. The recombinant protein in the soluble fraction was purified with Ni-NTA beads (Qiagen). The His-BVRF1 protein was eluted with elution buffer (50 mM NaH_2_PO_4_, 30 mM NaCl [pH 8.0], 250 mM imidazole). For mouse immunization, 30 μg of purified His-BVRF1 was mixed with the same volume of complete Freund’s adjuvant (Sigma) by vortexing for 10 min and injected subcutaneously to female BALB/c mice. The mice were then boosted with same amounts of antigen every 2 weeks. Antisera were collected and verified for antibody titers with 40 ng/mL tetradecanoylphorbol acetate (TPA) and 3 mM sodium butyrate (SB)-treated TW01-EBV cell lysates.

### Transfection.

TW01-EBV or HeLa cells were transfected with Lipofectamine 2000 (LF 2000; Invitrogen Co.) following the manufacturer’s instructions. Plasmids were transfected with Lipofectamine 2000 at a plasmid DNA (μg)Lipofectamine (μL) ratio of 2:3. The medium was refreshed at 6 h posttransfection, and the cells were incubated at 37°C with 5% CO_2_ for the indicated time. For rM81 virus production, 293/rM81 cells were transfected with TransIT-LT1 at a plasmid (μg)/TransIT-LT1 (μL) ratio of 1:3. The medium was refreshed at 16 hpt, and the cells were incubated at 37°C with 5% CO_2_ for the indicated time.

In the analysis of importin-β-mediated nuclear transport, 2 × 10^6^ HeLa cells were seeded onto silane coating microslides (no. SM5116, Muto Pure Chemicals Co., Ltd.) in 10-cm petri dishes and incubated for 24 h before transfection. Cells were transfected with 10 μg pEYFP-LacZ-serial plasmids by using TransIT-LT1 (Mirus Bio) according to the manufacture’s instructions. Then, 6 h posttransfection, culture medium was replaced by fresh 10% fetal bovine serum (FBS)-DMEM with or without IPZ, and the cells were incubated for another 18 h. Cells on slides were then fixed with 4% paraformaldehyde-PBS at room temperature (RT) for 20 min, washed with PBS 3 times, and subjected to immunofluorescence analysis.

### Western blotting.

TW01-EBV, HeLa, or 293/rM81 cells were harvested by scraping in phosphate-buffered saline (PBS; 0.8% NaCl, 0.02% KCl, 0.144% Na_2_HPO_4_, 0.024% KH_2_PO_4_, adjusted to pH 7.2), followed by centrifugation to harvest the cell pellets and lysis in RIPA buffer (1% NP-40, 50 mM Tris-HCl [pH 7.5], 150 mM NaCl, 0.5% deoxycholate, 0.1% SDS with 1 mM Na_3_VO_4_, 50 mM NaF, and 1× protease inhibitor (no. 11 873 580 001, Roche) and resolved by SDS-PAGE). Immunoblotting was performed as previously described (60) with Plus-ECL substrate (no. NEL103E001EA, Perkin Elmer).

### Immunofluorescence assay.

TW01-EBV or HeLa cells were seeded on glass slides in 10-cm dishes. After treatment or transfection, the slides were harvested at the indicated time points and washed with PBS, followed by fixation with 4% paraformaldehyde for 20 min at room temperature. Cells were washed with PBS for 5 min 3 times and permeabilized with 0.4% Triton X-100 in PBS for 5 min, followed by another 3 times of 5-min PBS washes. The cells on slides were then blocked with 1% BSA in PBS for 30 min and incubated with primary antibodies at 37°C for 1.5 h or at 4°C overnight followed by 3 times of 5-min PBS washes at room temperature. The slides were then incubated with secondary antibody at 37°C for 1.5 h, washed 3 times with PBS, and stained with Hoechst 33258 (Sigma; 1:5,000 in PBS) at room temperature for 5 min. With the mounting medium (H-1000, Vector Laboratories) covering, the slides were observed under a fluorescence microscope or confocal microscope (Zeiss AxioImager.Z2, LSM880 and LSM780, Cell Imaging Core of the First Core Labs, National Taiwan University College of Medicine).

### Virion purification and analysis.

The virus purification protocol was previously established in our laboratory (24). B95-8 cells were cultured in 400 mL of RPMI at saturated density and treated with 40 ng/mL TPA and 3 mM SB for 72 h. Cell pellets were removed by centrifuging the culture medium at 8,600 × *g* for 30 min at 4°C. The supernatant was then centrifuged at 20,000 × *g* for 1.5 h at 4°C using a JLA-16.250 rotor (Beckman) to pellet the viral particles. The pellet was then resuspended in TNE buffer (500 mM NaCl, 1 mM EDTA, 20 mM Tris-HCl [pH 7.4]) and layered on 25% to 65% (wt/vol) discontinuous sucrose-TNE gradient followed by ultracentrifugation with an SW41 Ti rotor (Beckman) at 25,000 × *g* for 4.5 h at 4°C (brake low). The solution was collected from top to bottom with each fraction of 0.9 mL and kept at 4°C before analysis. The virions in each fraction were detected with qPCR. In brief, a 49-μL aliquot of each fraction was mixed with 2 μL DNase I (1 u/μL) (Thermo Fisher, EN0521) and 0.2 μL 10× reaction buffer and then incubated at 37°C for 1 h, followed by heat inactivation at 70°C for 30 min to remove virion-free DNA. The samples were then treated with buffer containing 4 mM EDTA, 0.1% SDS, and 5 μg proteinase K and incubated at 37°C overnight to digest viral proteins. The viral DNA was then extracted by phenol-chloroform extraction followed by ethanol precipitation. To analyze viral proteins in different fractions, 850 μL of each fraction was subjected to ultracentrifugation at 21,300 × *g* for at least 2 h at 4°C. The pellets were resuspended in 50 μL of TNE buffer and agitated at 750 rpm for 30 min. Then, 10 μL of each sample was applied to SDS-PAGE for Western blotting with specific antibodies. For detecting gp350/220 and gp110, cell lysates were prepared in sample buffer without reducing agent.

### Transmission electron microscopy for virus particles.

For TEM sample fixation, 20 μL (about 5 μg protein weight) of sucrose gradient fraction was mixed with an equal volume of 4% paraformaldehyde and incubated for 30 min at room temperature. Negative-glow discharge of a Formvar-carbon-coated grid was performed with a GloQube Plus glow discharge system (Quorum Technologies, UK), using 20 mA for 30 s at 0.25 mbar. Paraformaldehyde-fixed suspensions were placed on the grid and incubated for 5 min. The vesicle-attached grid was washed with double-distilled water (ddH_2_O) 3 times. The samples were stained by adding a drop of 1% uranyl acetate for 1 min and then washed with ddH_2_O 5 times. After drying for 4 h, samples were examined with a Tecnai G2 F20 transmission electron microscope (FEI, USA) at 200 kV.

### rM81 virus preparation and Raji superinfection.

For rM81 virus production, 293/rM81 was induced by transfection of BZLF1 expression plasmid (p509) and BALF4 expression plasmid (p2670). The medium was replaced with fresh RPMI medium at 16 h posttransfection. EBV viral supernatants were harvested after incubation for 4.5 days, and cell debris was removed by centrifuging at 300 × *g* for 10 min. The culture supernatants were then filtered with a 0.45-μm filter. The virus was stored at 4°C. For Raji superinfection, 7.5 × 10^4^ cells/well of Raji cells were seeded in 12-well plates and cultured with the same volume of rM81 EBV and incubated for 72 h.

### Flow cytometry.

After EBV superinfection of Raji cells, flow cytometry was used to analyze the infectivity of EBV by measuring the percentage of Raji cells which express GFP. Infected cells were harvested and resuspended in 1.5 mL of fixing buffer (2% FBS, 2 mM EDTA, and 0.5% paraformaldehyde in PBS) and then assessed by reading on the BD LSRFortessa flow cytometer (1st CORE, College of Medicine, National Taiwan University) with BD FACSDiva software.

### Mass spectrum analysis.

To identify possible BALF2-interacting proteins, pCMV-Flag-BALF2 or pCMV-tag2B was transfected into TW01-EBV cells with pSG5-Rta to induce EBV replication. At 48 h posttransfection, cells were washed 3 times with PBS. The cell pellets were lysed with NP-40 lysis buffer (1% NP-40, 50 mM Tris, pH 8.0, 150 mM NaCl, 2 mM EDTA, and 1 mM Na_3_VO_4_) at 3 rpm and 4°C for 2 h. After genomic DNA fragmentation by sonication, the lysates were precleared by incubation with protein A Mag Sepharose beads (GE Healthcare, 28-9440-06) with rotation at 4°C for 40 min. Precleared lysates were incubated with anti-Flag-M2 antibody (Sigma, F3165) at 4°C for 18 h. The Flag-antibody immune-complexes were precipitated by incubation with protein A Mag Sepharose beads at 4°C and 3 rpm for 4 h. The beads were washed with NP-40 lysis buffer at 4°C, with 2 additional washes with PBS at 4°C. Beads were resuspended in the protein sample buffer and boiled at 95°C for 10 min for SDS-PAGE analysis and subjected to the in-gel digestion procedure to prepare tryptic peptides for the mass spectrometric analysis. Liquid chromatography tandem mass spectrometry (LC-MS/MS) analysis was performed on an Orbitrap Fusion mass spectrometry (Thermo Scientific, USA) equipped with a nano-electrospray ion source (New Objective, Woburn, MA). The liquid chromatographic separation was performed on a self-packed reversed-phase C_18_ capillary column (75 μm interior diameter [i.d.] by 200 mm, 3 μm, 100 Å) at a flow rate of 300 nL/min using 0.1% formic acid in water as mobile phase A and 0.1% formic acid in 80% acetonitrile as mobile phase B. The MS/MS RAW files were converted to mgf format using MSConvert (v3.0.9134; ProteoWizard) and subjected to Mascot (v2.3; Matrix Science, USA) for MS/MS ion search. The protein sequence database of Homo sapiens and was obtained from UniProt for MS/MS data analysis and the detailed protein annotation. The search parameters included an error tolerance for precursor ions and the MS/MS fragment ions in spectra of 10 ppm and 0.6 Da, respectively. The enzyme cutting site was set at the C-terminal of lysine and arginine with two missed cleavages. The variable posttranslational modifications in the search parameters were assigned to include oxidation of methionine and carbamidomethylation of cysteine. Peptides were identified with a false-discovery rate (FDR) of <1%.

### Coimmunoprecipitation assay.

TW01-EBV cells were seeded in 10-cm petri dish and cotransfected with plasmids expressing GFP-Rab1A and Rta or vector controls with TransIT-LT1 transfection reagent (Mirus, MIR2304) following the manufacturer’s manual. At 48 h posttransfection (hpt), cell pellets were lysed with 1 mL RIPA lysis buffer mix (1% NP-40, 50 mM Tris-HCl, pH 7.5, 150 mM NaCl, 0.5% deoxycholate, 0.1% SDS with 1 mM Na_3_VO_4_, 50 mM NaF and 1× protease inhibitor) at 4°C with rotation at for 2 h. Precleared lysate containing 500 μg protein was subjected to immunoprecipitation with 1 μg anti-BALF2 (OT13B) or anti-GFP (GTX113617, GeneTex) at 4°C overnight. The immunocomplexes were collected and washed twice with NP-40 lysis buffer (1% NP-40, 150 mM NaCl, 2 mM EDTA, 50 mM Tris-HCl, pH 8.0, with 1× protease inhibitor) and once with PBS. All the precipitates were subjected to SDS-PAGE, and precleared lysates containing 10 μg of protein were loaded as 2% input.

### Intracellular DNA isolation.

TW01-EBV or 293/rM81 cells were harvested and lysed with digestion buffer (50 mM Tris-HCl, pH 8.0, 0.125 mg/mL proteinase K, 0.1% SDS, 10 mM EDTA, pH 8.0) and incubated at 55°C for 3.5 h. The cell pellets were passed through 20-gauge needles 20 times. The lysates were supplemented with 0.5 mg/mL of RNase A and incubated at 55°C overnight. The DNA was purified with phenol-chloroform extraction followed by ethanol precipitation and resuspended in H_2_O.

### Virion DNA isolation.

The culture medium of TW01-EBV cells after reactivation was harvested and passed through a 0.45-μm polyethersulfone (PES) membrane (Millipore, SLHP033RS). Filtered culture medium (50 μL) was treated with 2 U DNase I (Thermo Fisher, EN0521) per reaction at 37°C for 1 h, followed by 70°C heat inactivation for 30 min to remove virion-free DNA. The sample was then treated with 50 μg/mL protease K at 50°C for 1 h, followed by 75°C heat inactivation for 30 min.

### Quantitative PCR.

The qPCR protocol was described previously (62). Briefly, the samples were quantified using PCR-coupled SYBR green I dye (Invitrogen) to detect the EBV BamHI W fragment or BALF5 (the primers were forward 5′-CCCTGTTTATCCGATGGAATG-3′ and reverse 5′-GGGTGACGAGGATGGAAA-3′) and human β globin (HBG). The standard curve for qPCR was generated by a 10-fold serial dilution of a mixture of 10^4^ copies of genomic DNA of 293TetER cells and 5 × 10^6^ copies of purified EBV bacmid DNA.

## References

[1] Johannsen E, Luftig M, Chase MR, Weicksel S, Cahir-McFarland E, Illanes D, Sarracino D, Kieff E. 2004. Proteins of purified Epstein-Barr virus. Proc Natl Acad Sci USA 101:16286–16291. doi:10.1073/pnas.0407320101. 15534216 PMC528973

[2] Lee MA, Diamond ME, Yates JL. 1999. Genetic evidence that EBNA-1 is needed for efficient, stable latent infection by Epstein-Barr virus. J Virol 73:2974–2982. doi:10.1128/JVI.73.4.2974-2982.1999. 10074147 PMC104057

[3] Seo J, Cho N, Kim H, Tsurumi T, Jang Y, Lee W, Lee S. 2008. Cell cycle arrest and lytic induction of EBV-transformed B lymphoblastoid cells by a histone deacetylase inhibitor, Trichostatin A. Oncol Rep 19:93–98. doi:10.3892/or.19.1.93. 18097580

[4] Tsai P-F, Lin S-J, Weng P-L, Tsai S-C, Lin J-H, Chou Y-C, Tsai C-H. 2011. Interplay between PKCδ and Sp1 on histone deacetylase inhibitor-mediated Epstein-Barr virus reactivation. J Virol 85:2373–2385. doi:10.1128/JVI.01602-10. 21159880 PMC3067761

[5] Grogan E, Jenson H, Countryman J, Heston L, Gradoville L, Miller G. 1987. Transfection of a rearranged viral DNA fragment, WZhet, stably converts latent Epstein-Barr viral infection to productive infection in lymphoid cells. Proc Natl Acad Sci USA 84:1332–1336. doi:10.1073/pnas.84.5.1332. 3029778 PMC304422

[6] Ragoczy T, Heston L, Miller G. 1998. The Epstein-Barr virus Rta protein activates lytic cycle genes and can disrupt latency in B lymphocytes. J Virol 72:7978–7984. doi:10.1128/JVI.72.10.7978-7984.1998. 9733836 PMC110133

[7] Fixman ED, Hayward GS, Hayward SD. 1995. Replication of Epstein-Barr virus oriLyt: lack of a dedicated virally encoded origin-binding protein and dependence on Zta in cotransfection assays. J Virol 69:2998–3006. doi:10.1128/JVI.69.5.2998-3006.1995. 7707526 PMC188999

[8] El-Guindy A, Ghiassi-Nejad M, Golden S, Delecluse H-J, Miller G. 2013. Essential role of Rta in lytic DNA replication of Epstein-Barr virus. J Virol 87:208–223. doi:10.1128/JVI.01995-12. 23077295 PMC3536415

[9] Lu C-C, Huang H-T, Wang J-T, Slupphaug G, Li T-K, Wu M-C, Chen Y-C, Lee C-P, Chen M-R. 2007. Characterization of the uracil-DNA glycosylase activity of Epstein-Barr virus BKRF3 and its role in lytic viral DNA replication. J Virol 81:1195–1208. doi:10.1128/JVI.01518-06. 17108049 PMC1797537

[10] Su M-T, Liu I-H, Wu C-W, Chang S-M, Tsai C-H, Yang P-W, Chuang Y-C, Lee C-P, Chen M-R. 2014. Uracil DNA glycosylase BKRF3 contributes to Epstein-Barr virus DNA replication through physical interactions with proteins in viral DNA replication complex. J Virol 88:8883–8899. doi:10.1128/JVI.00950-14. 24872582 PMC4136257

[11] Chang C-W, Lee C-P, Su M-T, Tsai C-H, Chen M-R. 2015. BGLF4 kinase modulates the structure and transport preference of the nuclear pore complex to facilitate nuclear import of Epstein-Barr virus lytic proteins. J Virol 89:1703–1718. doi:10.1128/JVI.02880-14. 25410863 PMC4300756

[12] Kalejta RF. 2008. Functions of human cytomegalovirus tegument proteins prior to immediate early gene expression, p 101–115. *In* Shenk TE, Stinski MF (ed), Human cytomegalovirus. Springer, Berlin, Germany. doi:10.1007/978-3-540-77349-8_6. 18637502

[13] Kelly BJ, Fraefel C, Cunningham AL, Diefenbach RJ. 2009. Functional roles of the tegument proteins of herpes simplex virus type 1. Virus Res 145:173–186. doi:10.1016/j.virusres.2009.07.007. 19615419

[14] Zhang K, van Drunen Littel-van den Hurk S. 2017. Herpesvirus tegument and immediate early proteins are pioneers in the battle between viral infection and nuclear domain 10-related host defense. Virus Res 238:40–48. doi:10.1016/j.virusres.2017.05.023. 28583441

[15] Owen DJ, Crump CM, Graham SC. 2015. Tegument assembly and secondary envelopment of alphaherpesviruses. Viruses 7:5084–5114. doi:10.3390/v7092861. 26393641 PMC4584305

[16] Radtke K, Kieneke D, Wolfstein A, Michael K, Steffen W, Scholz T, Karger A, Sodeik B. 2010. Plus- and minus-end directed microtubule motors bind simultaneously to herpes simplex virus capsids using different inner tegument structures. PLoS Pathog 6:e1000991. doi:10.1371/journal.ppat.1000991. 20628567 PMC2900298

[17] Ahmad I, Wilson DW. 2020. HSV-1 cytoplasmic envelopment and egress. Int J Mol Sci 21:5969. doi:10.3390/ijms21175969. 32825127 PMC7503644

[18] Nanbo A, Noda T, Ohba Y. 2018. Epstein-Barr virus acquires its final envelope on intracellular compartments with Golgi markers. Front Microbiol 9:454. doi:10.3389/fmicb.2018.00454. 29615992 PMC5864893

[19] Chiu YF, Sugden B, Chang PJ, Chen LW, Lin YJ, Lan YC, Lai CH, Liou JY, Liu ST, Hung CH. 2012. Characterization and intracellular trafficking of Epstein-Barr virus BBLF1, a protein involved in virion maturation. J Virol 86:9647–9655. doi:10.1128/JVI.01126-12. 22740416 PMC3446546

[20] Calderwood MA, Venkatesan K, Xing L, Chase MR, Vazquez A, Holthaus AM, Ewence AE, Li N, Hirozane-Kishikawa T, Hill DE, Vidal M, Kieff E, Johannsen E. 2007. Epstein-Barr virus and virus human protein interaction maps. Proc Natl Acad Sci USA 104:7606–7611. doi:10.1073/pnas.0702332104. 17446270 PMC1863443

[21] Gulbahce N, Yan H, Dricot A, Padi M, Byrdsong D, Franchi R, Lee D-S, Rozenblatt-Rosen O, Mar JC, Calderwood MA, Baldwin A, Zhao B, Santhanam B, Braun P, Simonis N, Huh K-W, Hellner K, Grace M, Chen A, Rubio R, Marto JA, Christakis NA, Kieff E, Roth FP, Roecklein-Canfield J, Decaprio JA, Cusick ME, Quackenbush J, Hill DE, Münger K, Vidal M, Barabási A-L. 2012. Viral perturbations of host networks reflect disease etiology. PLoS Comput Biol 8:e1002531. doi:10.1371/journal.pcbi.1002531. 22761553 PMC3386155

[22] Procter DJ, Banerjee A, Nukui M, Kruse K, Gaponenko V, Murphy EA, Komarova Y, Walsh D. 2018. The HCMV assembly compartment is a dynamic Golgi-derived MTOC that controls nuclear rotation and virus spread. Dev Cell 45:83–100.e7. doi:10.1016/j.devcel.2018.03.010. 29634939 PMC5896778

[23] Hung CH, Chiu YF, Wang WH, Chen LW, Chang PJ, Huang TY, Lin YJ, Tsai WJ, Yang CC. 2019. Interaction between BGLF2 and BBLF1 is required for the efficient production of infectious Epstein-Barr virus particles. Front Microbiol 10:3021. doi:10.3389/fmicb.2019.03021. 32038519 PMC6993569

[24] Wang J-T, Yang P-W, Lee C-P, Han C-H, Tsai C-H, Chen M-R. 2005. Detection of Epstein-Barr virus BGLF4 protein kinase in virus replication compartments and virus particles. J Gen Virol 86:3215–3225. doi:10.1099/vir.0.81313-0. 16298966

[25] Kaku N, Matsuda KI, Tsujimura A, Kawata M. 2008. Characterization of nuclear import of the domain-specific androgen receptor in association with the importin alpha/beta and Ran-guanosine 5′-triphosphate systems. Endocrinology 149:3960–3969. doi:10.1210/en.2008-0137. 18420738 PMC2488236

[26] Alvarez C, Garcia-Mata R, Brandon E, Sztul E. 2003. COPI recruitment is modulated by a Rab1b-dependent mechanism. Mol Biol Cell 14:2116–2127. doi:10.1091/mbc.e02-09-0625. 12802079 PMC165101

[27] Saraste J. 2016. Spatial and functional aspects of ER-Golgi Rabs and tethers. Front Cell Dev Biol 4:28. doi:10.3389/fcell.2016.00028. 27148530 PMC4834429

[28] Bertoni G, Nguyen QV, Humphreys RE, Sairenji T. 1989. Intracellular synthesis of Epstein-Barr virus membrane antigen gp350/220. Intervirology 30:61–73. doi:10.1159/000150077. 2542183

[29] Yang J-S, Valente C, Polishchuk RS, Turacchio G, Layre E, Branch MD, Leslie CC, Gelb MH, Brown WJ, Corda D, Luini A, Hsu VW. 2011. COPI acts in both vesicular and tubular transport. Nat Cell Biol 13:996–1003. doi:10.1038/ncb2273. 21725317 PMC3149785

[30] Klausner RD, Donaldson JG, Lippincott-Schwartz J. 1992. Brefeldin A: insights into the control of membrane traffic and organelle structure. J Cell Biol 116:1071–1080. doi:10.1083/jcb.116.5.1071. 1740466 PMC2289364

[31] Whealy ME, Card JP, Meade RP, Robbins AK, Enquist LW. 1991. Effect of brefeldin A on alphaherpesvirus membrane protein glycosylation and virus egress. J Virol 65:1066–1081. doi:10.1128/JVI.65.3.1066-1081.1991. 1847436 PMC239872

[32] Tsai MH, Raykova A, Klinke O, Bernhardt K, Gartner K, Leung CS, Geletneky K, Sertel S, Munz C, Feederle R, Delecluse HJ. 2013. Spontaneous lytic replication and epitheliotropism define an Epstein-Barr virus strain found in carcinomas. Cell Rep 5:458–470. doi:10.1016/j.celrep.2013.09.012. 24120866

[33] Kawashima D, Kanda T, Murata T, Saito S, Sugimoto A, Narita Y, Tsurumi T. 2013. Nuclear transport of Epstein-Barr virus DNA polymerase is dependent on the BMRF1 polymerase processivity factor and molecular chaperone Hsp90. J Virol 87:6482–6491. doi:10.1128/JVI.03428-12. 23552409 PMC3648106

[34] Milbradt J, Sonntag E, Wagner S, Strojan H, Wangen C, Lenac Rovis T, Lisnic B, Jonjic S, Sticht H, Britt WJ, Schlötzer-Schrehardt U, Marschall M. 2018. Human cytomegalovirus nuclear capsids associate with the core nuclear egress complex and the viral protein kinase pUL97. Viruses 10:35. doi:10.3390/v10010035. 29342872 PMC5795448

[35] Saraste J, Marie M. 2018. Intermediate compartment (IC): from pre-Golgi vacuoles to a semi-autonomous membrane system. Histochem Cell Biol 150:407–430. doi:10.1007/s00418-018-1717-2. 30173361 PMC6182704

[36] Marra P, Maffucci T, Daniele T, Tullio GD, Ikehara Y, Chan EK, Luini A, Beznoussenko G, Mironov A, De Matteis MA. 2001. The GM130 and GRASP65 Golgi proteins cycle through and define a subdomain of the intermediate compartment. Nat Cell Biol 3:1101–1113. doi:10.1038/ncb1201-1101. 11781572

[37] Barrowman J, Bhandari D, Reinisch K, Ferro-Novick S. 2010. TRAPP complexes in membrane traffic: convergence through a common Rab. Nat Rev Mol Cell Biol 11:759–763. doi:10.1038/nrm2999. 20966969

[38] Johns HL, Gonzalez-Lopez C, Sayers CL, Hollinshead M, Elliott G. 2014. Rab6 dependent post-Golgi trafficking of HSV1 envelope proteins to sites of virus envelopment. Traffic 15:157–178. doi:10.1111/tra.12134. 24152084 PMC4345966

[39] Zenner HL, Yoshimura S-i, Barr FA, Crump CM. 2011. Analysis of Rab GTPase-activating proteins indicates that Rab1a/b and Rab43 are important for herpes simplex virus 1 secondary envelopment. J Virol 85:8012–8021. doi:10.1128/JVI.00500-11. 21680502 PMC3147948

[40] Sklan EH, Serrano RL, Einav S, Pfeffer SR, Lambright DG, Glenn JS. 2007. TBC1D20 is a Rab1 GTPase-activating protein that mediates hepatitis C virus replication. J Biol Chem 282:36354–36361. doi:10.1074/jbc.M705221200. 17901050

[41] Nachmias D, Sklan EH, Ehrlich M, Bacharach E. 2012. Human immunodeficiency virus type 1 envelope proteins traffic toward virion assembly sites via a TBC1D20/Rab1-regulated pathway. Retrovirology 9:7. doi:10.1186/1742-4690-9-7. 22260459 PMC3283470

[42] Jowers TP, Featherstone RJ, Reynolds DK, Brown HK, James J, Prescott A, Haga IR, Beard PM. 2015. RAB1A promotes Vaccinia virus replication by facilitating the production of intracellular enveloped virions. Virology 475:66–73. doi:10.1016/j.virol.2014.11.007. 25462347 PMC4292983

[43] Lin J, Wang C, Liang W, Zhang J, Zhang L, Lv H, Dong W, Zhang Y. 2018. Rab1A is required for assembly of classical swine fever virus particle. Virology 514:18–29. doi:10.1016/j.virol.2017.11.002. 29128753

[44] Seto S, Tsujimura K, Koide Y. 2011. Rab GTPases regulating phagosome maturation are differentially recruited to mycobacterial phagosomes. Traffic 12:407–420. doi:10.1111/j.1600-0854.2011.01165.x. 21255211

[45] Junutula JR, De Maziére AM, Peden AA, Ervin KE, Advani RJ, van Dijk SM, Klumperman J, Scheller RH. 2004. Rab14 is involved in membrane trafficking between the Golgi complex and endosomes. Mol Biol Cell 15:2218–2229. doi:10.1091/mbc.e03-10-0777. 15004230 PMC404017

[46] Morimoto S, Nishimura N, Terai T, Manabe S, Yamamoto Y, Shinahara W, Miyake H, Tashiro S, Shimada M, Sasaki T. 2005. Rab13 mediates the continuous endocytic recycling of occludin to the cell surface. J Biol Chem 280:2220–2228. doi:10.1074/jbc.M406906200. 15528189

[47] Simpson JC, Griffiths G, Wessling-Resnick M, Fransen JA, Bennett H, Jones AT. 2004. A role for the small GTPase Rab21 in the early endocytic pathway. J Cell Sci 117:6297–6311. doi:10.1242/jcs.01560. 15561770

[48] Köhler K, Louvard D, Zahraoui A. 2004. Rab13 regulates PKA signaling during tight junction assembly. J Cell Biol 165:175–180. doi:10.1083/jcb.200312118. 15096524 PMC2172036

[49] Xu D, Li Y, Wu L, Li Y, Zhao D, Yu J, Huang T, Ferguson C, Parton RG, Yang H, Li P. 2018. Rab18 promotes lipid droplet (LD) growth by tethering the ER to LDs through SNARE and NRZ interactions. J Cell Biol 217:975–995. doi:10.1083/jcb.201704184. 29367353 PMC5839781

[50] Hung C-H, Chen L-W, Wang W-H, Chang P-J, Chiu Y-F, Hung C-C, Lin Y-J, Liou J-Y, Tsai W-J, Hung C-L, Liu S-T. 2014. Regulation of autophagic activation by Rta of Epstein-Barr Virus via the extracellular signal-regulated kinase pathway. J Virol 88:12133–12145. doi:10.1128/JVI.02033-14. 25122800 PMC4178756

[51] Granato M, Santarelli R, Farina A, Gonnella R, Lotti LV, Faggioni A, Cirone M. 2014. Epstein-Barr virus blocks the autophagic flux and appropriates the autophagic machinery to enhance viral replication. J Virol 88:12715–12726. doi:10.1128/JVI.02199-14. 25142602 PMC4248894

[52] Lin CT, Chan WY, Chen W, Huang HM, Wu HC, Hsu MM, Chuang SM, Wang CC. 1993. Characterization of seven newly established nasopharyngeal carcinoma cell lines. Lab Invest 68:716–727. 7685844

[53] Shimizu N, Yoshiyama H, Takada K. 1996. Clonal propagation of Epstein-Barr virus (EBV) recombinants in EBV-negative Akata cells. J Virol 70:7260–7263. doi:10.1128/JVI.70.10.7260-7263.1996. 8794379 PMC190785

[54] Sarisky RT, Gao Z, Lieberman PM, Fixman ED, Hayward GS, Hayward SD. 1996. A replication function associated with the activation domain of the Epstein-Barr virus Zta transactivator. J Virol 70:8340–8347. doi:10.1128/JVI.70.12.8340-8347.1996. 8970953 PMC190921

[55] Sannerud R, Marie M, Nizak C, Dale HA, Pernet-Gallay K, Perez F, Goud B, Saraste J. 2006. Rab1 defines a novel pathway connecting the pre-Golgi intermediate compartment with the cell periphery. Mol Biol Cell 17:1514–1526. doi:10.1091/mbc.e05-08-0792. 16421253 PMC1415313

[56] Nevo-Yassaf I, Yaffe Y, Asher M, Ravid O, Eizenberg S, Henis YI, Nahmias Y, Hirschberg K, Sklan EH. 2012. Role for TBC1D20 and Rab1 in hepatitis C virus replication via interaction with lipid droplet-bound nonstructural protein 5A. J Virol 86:6491–6502. doi:10.1128/JVI.00496-12. 22491470 PMC3393552

[57] Ishida M, Ohbayashi N, Fukuda M. 2015. Rab1A regulates anterograde melanosome transport by recruiting kinesin-1 to melanosomes through interaction with SKIP. Sci Rep 5:8238. doi:10.1038/srep08238. 25649263 PMC4316160

[58] Zeng Y, Middeldorp J, Madjar JJ, Ooka T. 1997. A major DNA binding protein encoded by BALF2 open reading frame of Epstein-Barr virus (EBV) forms a complex with other EBV DNA-binding proteins: DNAase, EA-D, and DNA polymerase. Virology 239:285–295. doi:10.1006/viro.1997.8891. 9434720

[59] Hsu T-Y, Chang Y, Wang P-W, Liu M-Y, Chen M-R, Chen J-Y, Tsai C-H. 2005. Reactivation of Epstein–Barr virus can be triggered by an Rta protein mutated at the nuclear localization signal. J Gen Virol 86:317–322. doi:10.1099/vir.0.80556-0. 15659750

[60] Chen M-R, Chang S-J, Huang H, Chen J-Y. 2000. A protein kinase activity associated with Epstein-Barr virus BGLF4 phosphorylates the viral early antigen EA-D in vitro. J Virol 74:3093–3104. doi:10.1128/jvi.74.7.3093-3104.2000. 10708424 PMC111808

[61] Kishishita M, Luka J, Vroman B, Poduslo JF, Pearson GR. 1984. Production of monoclonal antibody to a late intracellular Epstein-Barr virus-induced antigen. Virology 133:363–375. doi:10.1016/0042-6822(84)90402-1. 6324457

[62] Su M-T, Wang Y-T, Chen Y-J, Lin S-F, Tsai C-H, Chen M-R. 2017. The SWI/SNF chromatin regulator BRG1 modulates the transcriptional regulatory activity of the Epstein-Barr virus DNA polymerase processivity factor BMRF1. J Virol 91:e02114-16. doi:10.1128/JVI.02114-16. 28228591 PMC5391464

